# The Complicity of DAAM1, PTMA, RSPH6A, and Steroidogenic Genes in the Fertility of Male Rats Exposed to Cadmium During Gestation and Lactation: Attenuation by PREOG

**DOI:** 10.1007/s43032-025-01902-x

**Published:** 2025-07-08

**Authors:** Ikokide Emmanuel Joseph, Jaja Ishmael Festus, Temitayo Olabisi Ajibade, Ademola Adetokunbo Oyagbemi, Mathew Olugbenga Oyeyemi

**Affiliations:** 1 Department of Theriogenology, Faculty of Veterinary Medicine, University of Ibadan, Ibadan, Oyo State Nigeria; 2 Department of Livestock and Pasture Science, University of Fort Hare, Alice, 5700 South Africa; 3 Department of Agriculture and Animal Health, University of South Africa, Roodepoort, Johannesburg 1710 South Africa; 4 Department of Veterinary Physiology and Biochemistry, Faculty of Veterinary Medicine, University of Ibadan, Ibadan, Oyo State Nigeria

**Keywords:** Cadmium, PREOG, q-RT-PCR, Steroidogenesis, PTMA. RSPH6A, DAAM1

## Abstract

The role of cadmium in the dwindling male fertility rate in men and animals has been extensively documented in several studies over the years, and various natural compounds have been used to successfully manage it. In this study, we investigated the impact of exposing dams to polyphenol-rich extract of *Ocimum gratissimum* (PREOG)/cadmium during gestation and lactation on the reproductive outcome in adult male offspring (PND 90). Sixty adult females were randomly assigned to groups A-F (*n* = 10) and cohabitated with adult males in a ratio of 2:1 for ten days. At day 7, each female received oral treatment daily as follows; A (served as the control), B (received 3 mg/kg CdCL_2_), C (received 100 mg/kg PREOG), D (received 200 mg/kg PREOG), E (received 100 mg/kg PREOG + 3 mg/kg CdCL_2_) and F (received 200 mg/kg PREOG + 3 mg/kg CdCL_2_) treatment continued to postnatal day 29 before weaning. The male pups separated from females continued to receive only food and water *ad lib* till postnatal ninety (PND90). At (PND 90) five males were selected from each group cohabited with the untreated females at a ratio of 2:1 for a fertility test, while six males from each were sacrificed, and blood, semen, and testes were harvested for analysis. Our result indicated that PREOG enhanced semen quality, GSI/EPI, SOD, GPx, and GST values at (PND90), also PREOG upregulates the expression of StAR, 3β-HSD, 17β-HSD genes, and testosterone value in (PND90), PREOG also upregulated the expression of PTMA, RSPH6A, DAAM1 genes, and the fertility outcome in (PND90) offspring. These findings validate the protective role of PREOG against cadmium-induced reprotoxcity in male offspring exposed during gestation and lactation and enhance reproductive performance.

**Graphical Abstract:**

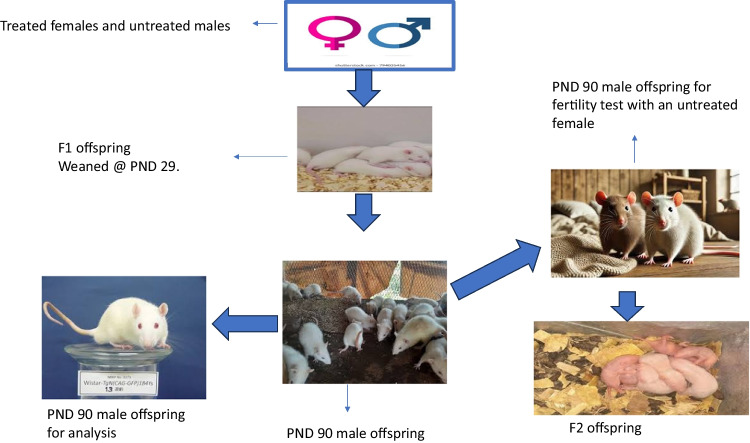

**Supplementary Information:**

The online version contains supplementary material available at 10.1007/s43032-025-01902-x.

## Background

The reproductive outcome of childlessness is called infertility. Male infertility could be of primary or acquired origin, copulatory or fertilization problems, and anything that can impair male reproductive capacity [1, 2]. In bulls, Infertility is the inability to secure pregnancies, whereas sub-fertility (i.e. reduced fertility) is impaired conception, an extension of calving season, decreased weaning weights of a calf, and an elevated number of culled females, consequently, there will be economic losses and threats in sustaining the livestock operation [3]. Recently, the universal decline in male fertility in humans and animals pre-empted reasoning as to what could be the link between the decrease in sperm quality and its relationship with universal changes [4]. A meta-analysis in humans documented a 50% decline in sperm counts of men in Western cultures in the past 40 years [5]. Thippeswamy et al. reported that in crossbred bulls, there has been a decline in the acceptable quality semen-producing ability from generation to generation [6].

Defects and abnormalities of the spermatozoa are complicit to the increased incidence of male infertility situation in men and animals. The diagnosis and management of spermatogenic failure continue to thrill medical practitioners and veterinarians despite significant progress in the investigation and management of infertility cases [7, 8], and of the numerous procedures used in diagnosing spermatogenic failure, several cases remain undiagnosed and declared as idiopathic. Seasonal variation, heterogeneous population of sperm within a single ejaculate, the influence of semen freezing procedure, etc., further add to the disparity in results [9]. Recently, the causative factors or etiology that culminate in the decline of the male fertility rate has been associated with a common intrinsic mechanism of impairing the intricate balance between the production and scavenging of reactive oxygen species (ROS) shared by most of these factors. This imbalance of increased ROS generation and reduced antioxidant capacity leads to oxidative stress (OS) [10]. Oxidative stress is a major threat spermatozoon must contend with, and in most cases of imbalance between free radicals (reacting oxygen or nitrogen species ROS) and antioxidant system, the oxidant is always favored [11]. Although, numerous physiological functions of the spermatozoa are regulated by redox metabolism, such as acrosome reaction (AR), capacitation, and sperm-oocyte interactions [12, 13], unlimited generation or high levels of free radicals are detrimental to all cell component and at molecular level, DNA and lipids are specifically vulnerable [12]. Additionally, in normal spermatozoa, oxidative stress may occur as a result of the presence of leukocytes that generate ROS in the semen, exposure to exogenous substances, spermatozoa mitochondria metabolism, and in extended semen for assisted reproductive technique (ART) [12].

It is no longer news that lifestyle and environmental intoxicants, in addition to other etiologies have been implicated as the major contributing factor in the dwindling male fertility rate [14]. One of these intoxicants with universal distribution is cadmium. Human activities such as the use of fossil fuels, copper alloys, and coverings to protect iron and steel from corrosion, cadmium is also an ingredient in paints used with plastic and ceramics, it is used to manufacture Nickel–Cadmium hydroxide batteries (Ni–Cd) for the automobile industry. Cadmium is abundant in phosphorus-based fertilizers and pesticides, it is also in run-off cultivated fields, as residue in mining sites, and in garbage dumps as leachates; thus, it is easily absorbed into animal feed and foods for human consumption, mainly as cadmium chloride (CdCl_2_), cadmium oxide (CdO) and cadmium sulfate (CdS) [15]. Cadmium can mimic vital physiological ions thus interfering with normal biological processes. One major way the reproductive system is distorted is by disrupting the endocrine system [16, 17], enhancing oxidative, apoptotic, and inflammatory processes [18, 19], and by impairing spermatogenesis [18, 20]. The detrimental impact of cadmium on the general well-being and fertility rate is well documented [21]. Due to its long half-live and non-degradable nature, cadmium amasses longer than expected in the body even at minimal exposure thus exerting its lethal effects. Pregnant and lactating animals have been shown to assimilate and retain more dietary cadmium than their non-pregnant counterpart, thus intoxicating developing fetuses and breastfeeding animals by maternal exposure [22]. It has also been documented that exposure to estrogen or estrogen likes (cadmium) elicits early puberty and impairs the development of mammary gland in female offspring [23] decreased testicular size and testosterone levels, and production of spermatozoa and enhances sub-fertility in male offspring [24]. More recent studies documented [25–27] also reported the detrimental effects of maternal exposure to cadmium on male offspring.

Polyphenolic-rich extract of *Ocimum gratissimum* (PREOG) is a fraction of *Ocimum gratissimum* rich in phytocompounds such as flavonoids, phenol, and tannin. Invitro assay of PREOG shows it a potent antioxidant with an IC_50_ below 50 [28, 29], and the therapeutic properties of *Ocimum gratissimum* can be associated with its rich phenolic content which has been documented to possess strong antioxidative property [30, 31]. The polyphenolic extract of *Ocimum gratissimum* has been used to manage several pathologic conditions in different study modules successfully [29, 32, 33]. In our previous study, we reported that dam exposed to cadmium during gestation and lactation results in impaired reproductive potential in prepubertal male offspring (PND30), the co-treatment with PREOG abrogated the cadmium effect. This current study is a continuation, to ascertain if the impaired reproductive potential prevented in prepubertal male offspring (PND30) of dam exposed to PREOG during gestation and lactation, translates to improved reproductive outcome at the pubertal or adult (PND90) age of the male offspring. Our focus is testicular biometry (gonadosomatic and epididymal index, left and right testicular diameter/length, histology of the testes), semen analysis, and serum hormonal. Moreover, testicular expression of StAR, 3β-HSD1 and 17β-HSD3 steroidogenic genes, RSPH6A gene associated with sperm motility, DAAM1 gene associated with the cytoskeleton of the sperm, PTMA gene associated with maintaining normal acrosome integrity and finally fertility study if the pubertal male (PND90) can sire an offspring.

## Material and Method

### Plant Collection and Identification

Freshly harvested leaves of *Ocimum gratissimum* were obtained from different farmers around Ibadan, Oyo State, Nigeria. The harvested plant was authenticated after identification at the Botany Department, University of Ibadan, Ibadan. A voucher specimen was deposited at the herbarium with reference number UIH-22617.

### Polyphenolic Extraction

The leaves were dried in a shaded area and made into their powdered form. The fat in the powder was removed by immersing it for 24 h in n-hexane. The nonfat powder is soaked in methanol for another 72 h to get the methanol crude extract. The crude methanol extract was concentrated using a rotatory evaporator at 40 °C. The sticky residue was then partitioned into chloroform soluble fractions using chloroform. The chloroform-rich fraction was then evaporated and dried in an oven under reduced pressure to obtain a polyphenolic fraction of *Ocimum gratissimum* (PREOG) [34].

### Reagents

Cadmium chloride was purchased from Loba chemicals as cadmium chloride monohydrate (98%) extra pure, CAS number 35658–65-2 was dissolved in sterile distilled water to a concentration of 3 mg/kg with slight modification [35]**.** The procedure as described by [34] was used to extract polyphenol-rich content from *Ocimum gratissimum* leaves and con oil was used to reconstitute it for oral administration. The concentrations 100 mg/kg and 200 mg/kg were chosen following the result we got from our toxicity study [36].

### Experimental Design

Sixty adult virgin female Wistar rats weighing 140 ± 10 g showing regular estrus cycle were randomly assigned to six groups A to F of ten rats each. They were allowed to acclimatize for two weeks. Thirty adults male Wistar rat weighing 220 ± 10 g were transferred to a mating cage and cohabited with female rats in the proestrus stage (2:1) for 10 days. Since the estrous length in rodents is about 4 to 5 days [37], the females were allowed to acclimatize for two as this will synchronize estrous naturally (Lee-Boot effects) before introduction to males, we started counting three days upon introduction of males to females as gestation day one (GSD1) (Whitten Effect) [38, 39]. At GSD 7, females started receiving treatment as follows, this is done to ensure that treatment commences before organogenesis [40]. Group A as a positive control was only allowed unlimited access to water supply and feed, group B negative control received 3 mg/kg CdCL_2_ orally, group C received 100 mg/kg PREOG only, group D received 200 mg/kg PREOG only, group E received 100 mg/kg PREOG + 3 mg/kg CdCL_2_ and group F received 200 mg/kg PREOG + 3 mg/kg CdCL_2_. The treatment groups were dosed daily from gestation seven (GSD7) post-natal day twenty-nine (PND 29). At PND 30, the pups were weaned from the dams, and the male pups were all grouped A to F on a unisexual litter basis. The weaned male pups continued to have unrestricted access to feed and water until PND90. At the PN90, six adult males were taken from each group and sacrificed, while blood, semen, and testes were collected for analysis.

#### Gonadosomatic and Epididymis Index

The weight of the left and right testes and epididymis were immediately measured using a sensitive electronic weighing machine. The GSI and EPI were computed as the average testicular weight (g) or epididymal weight (g) divided by the weight difference (at day one and sixty-five) (g) and multiplied by 100. GSI and EPI value is expressed in percentage (%) [41].

#### Motility

Sperm motility was determined using procedures described by [42] with slight modification by [43]. Semen was isolated from the caudal epididymis and placed in a warm slide. Using a warm sodium citrate buffer (2.9%) the semen was mixed, and a cover slip was then placed on the slide before microscopic evaluation. From isolation to examination is approximately 2–4 min and our result is expressed in percentage.

#### Livability

A smear was prepared from the caudal epididymal semen in a non-greasy slide and stained with Wells and Awa 1% Eosin and 5% Nigrosin in 2.9% sodium citrate dehydrates solution for the live/dead ration determination as demonstrated [43] with slight modification.

#### Sperm Count

Epididymal sperm count was evaluated by chopping up the cauda epididymis in distilled water and sieved with a nylon mesh. The spermatozoa were counted using Pant and Srivastava with improved Neubauer chamber technique as demonstrated [43] with slight modification.

#### Morphological Characteristics

A smear was made on a clean slide free of grease with semen collected from the caudal epididymis and stained with Awa and Wells stain as demonstrated by [43]. The smear was air-dried, and with the aid of a microscope the abnormal cells from several fields of at least 400 sperm cells were counted and their total percentage was determined.

#### Serum Hormonal Assay

Serum harvested were decanted into plain bottles for analyzing luteinizing hormone (LH), testosterone (T), and follicle-stimulating hormone (FSH) levels using standard Enzyme-linked immunosorbent assay (Monobind ELISA) kits (425–300 FSH AccuBind, 3725–300 Testosterone AccuBind, 675–300 LH AccuBind, 4825–300 Progesterone AccuBind, and GnRH-CEA843Mi Cloud-Clone Corp) using manufacturer’s guide.

#### Tissue Processing

##### Tissue Biochemical Assays

The testes using 50 mMTris– HCl buffer (pH 7.4) containing 1.15% potassium chloride were homogenized and the homogenate was centrifuged for 15 min at 12 000 rpm and 4 °C. For estimating biochemical parameters, the resulting supernatant was used. Total protein concentration was documented [44]. Hydrogen peroxide generation was as described [45]. The malondialdehyde (MDA) value was demonstrated as described [46]. Superoxide dismutase (SOD) activity was determined as previously described [47] with little modification [48]. Glutathione S-transferase (GST) was determined as documented by [49]. Glutathione peroxidase (GPx) activity was carried out as documented [50], where hydrogen peroxide was used as substrate. Reduced glutathione (GSH) was determined as documented [51] at 412 nm, with little modifications [48].

##### Quantitative Real-Time PCR

The extraction of total RNA from the testes of the experimental group was done using a modified CTAB extraction protocol and 20 ng of total RNA was treated with NEB DNase 1 (M0303) to eliminate extracted DNA. For the gene quantification, 20 µL reactions following the manufacturer’s instructions using Luna® Universal qPCR Master Mix Protocol (M3003) were used to identify the presence of StAR, 3β-HSD, 17β- HSD, PTMA, RSPH6A, and DAAM1 genes in the extracted RNA. Expression of the Actin gene was the internal control. Briefly, a mix of 10 µL Luna Universal qPCR Master Mix, 0.06 Reverse Transcriptase (Promega) made up to 18 µL with Nuclease-free Water to which 2 µL of the treated RNA Template and 0.5 µL Forward primer (10 µM) 0.5 µL Reverse primer (10 µM) was added. This was then run with the outline starting Denaturation for 60 s at 95 °C, then 40–45 of Denaturation for 15 s at 95 °C, plate reading, and Extension for 30 s at 60 °C, and termination for 10 min at 72 °C. Amplification was carried out with the cfx96tm real-time system from Bio-Rad using the manufacturer manual. The threshold cycle number (Ct value) was analyzed using the cfx96tm real-time system from Bio-Rad. QPCR was normalized to the Ct value of ACTB, from the same sample, and the fold changes in the expression of each gene were calculated by using the delta-delta Ct method. A non-template control was included in each experiment. Primer sequences used for quantitative real-time PCR of genes are indicated in the table below and are synthesized in South Africa at Inqaba Table 1.

**Table 1:** Properties of primer used

Genes	Forward primers	Reverse primers
RSPH6A	TGCAACCTCAGCCTGTATG	AGAGACTCCAGGATGGACAA
PTMA	GCCATCTTTGCATTGTTCCC	GGTGCTACGCGTAAGGAATTA
BHSD	CACTACGAAGTCGATGGTTCAG	CCTTCCAGGACATTGGCTAA
STAR	CAGCAGGAGAATGGAGATGAA	GGTCCACCAGTTCTTCATAGAG
B17HSD	CACCTCTTTGCCCACTATCA	TCGCATCGCAGTCAAGAA
DAAM1	GCCTTATCATCGCTCTCATCTT	GGTCCATGGTGTACACTTTCA
ACTIN	AGCCATGTACGTAGCCATCC	ACCCTCATAGATGGGCACAG

##### Histological Preparations

The testes were harvested from each male rat and fixed by immersion in Bouin’s fluid for 48 h. Afterward, they were dehydrated in different concentrations of ethanol, and xylene was used to clear before embedment in paraffin wax. A section of 5 μm thick was cut, mounted on glass slides, and stained with eosin and hematoxylin for light microscopic examination.

### Fertility Test

Two weeks before (PND90) 60 adult females albino Wistar rats weighing 150 ± 10 g were purchased and allowed to acclimatize. The remaining male offspring (PND90) of dams exposed to cadmium and PREOG during gestation and lactation were allowed to cohabit with the untreated females in a ratio of two females to a male (2:1) for ten days before the males were removed and the females kept in their separate’s cages until term. The percentage of fertility was calculated by considering the number of females bred and the number of females conceived. The pregnant females were allowed to deliver the pups. The number of females conceived from each group and the number of pups born were recorded and expressed as mean values of each group, the viability of pups from PND0 to PND7 was determined as the survival index for each litter, and the fertility indices were determined according to the procedure of [52, 53] with slight modification as follows:

#### Male Fertility Index

Fertility index = (Number of fertile males ÷ number of males used in the test) × 100

#### Female Fertility Index

Fertility index = (Number of pregnant rats ÷ number of mated females) × 100

#### Parturition Index

Parturition index = (Number of females delivered ÷ number of pregnant rats) × 100

#### Gestation Index

Gestation index = (Number of pups burn alive ÷ total number of pups burn) × 100

The number of pups delivered was expressed as the mean percentage of pups delivered during parturition.

### Statistical Analyses

The results of the experiment were expressed as mean ±SD. Differences between multiple groups were analyzed by one-way analysis of variance (ANOVA), followed by Student’s t-test was used to determine significant differences indicative of changes between the control and the treated groups with Graph Pad Prism 5. The results were considered statistically significant when values of *p* < 0.05.

## Result

### Gonadosomatic and Epididymal Index in Pubertal Offspring (PND90)

Table 2 shows testicular GSI and EPI of pubertal male offspring (PND90) exposed to cadmium, PREOG during gestation and lactation. At PND90 male offspring exposed to cadmium, and PREOG during gestation and lactation had a marked weight gain (*p* < 0.05) across all groups. But contrasted to control, weight gain was insignificant (*p* > 0.05) in cadmium group. Weight gains of the different doses of PREOG and cadmium were comparable to the cadmium and control group. However, in group E (222.97 ± 2.79) weight gain difference was notable (*p* < 0.05) to cadmium group B (222.29 ± 2.17), also in groups F (216.60 ± 4.36) weight gain was notably lower (*p* < 0.05) to control A (222.24 ± 2.42). Weight gain of the different doses of PREOG alone was notably different to control and cadmium groups. But in group C (219.24 ± 3.12) weight gain was comparable to control A (222.24 ± 2.42) and cadmium group B (222.29 ± 2.17) (Table 2).

**Table 2:** GSI and EPI of pubertal male offspring (PND90) exposed to PREOG/cadmium during gestation and lactation

Weight/GSI/ EPI/groups	Left & right testes average weight (g)	PND90 & PND1 weight difference (g)	Epididymal weight (g)	GSI (%)	EPI (%)
A	1.35 ± 0.34	222.24 ± 2.42	0.10 ± 0.07	0.61 ± 0.04	0.05 ± 0.003
B	0.95 ± 0.10^a^	222.29 ± 2.17	0.05 ± 0.01	0.43 ± 0.03^a^	0.02 ± 0.001^a^
C	1.35 ± 0.36^b^	219.24 ± 3.12	0.11 ± 0.07	0.62 ± 0.05^b^	0.05 ± 0.003^b^
D	1.25 ± 0.25^b^	213.49 ± 5.43^ab^	0.09 ± 0.05	0.59 ± 0.02^b^	0.04 ± 0.003^ab^
E	1.10 ± 0.10^b^	222.97 ± 2.79^b^	0.09 ± 0.04	0.49 ± 0.01^ab^	0.04 ± 0.002^ab^
F	1.05 ± 0.17	216.60 ± 4.36^a^	0.08 ± 0.01	0.49 ± 0.01^ab^	0.04 ± 0.001^ab^
*p*	0.0732	0.0014	0.4924	0.0001	0.0001

Values are express as mean ± SD (*n* = 5). p < 0.05 using one-way ANOVA is markedly different across all groups. Using student t-test. Superscript ^a b^ = (*p* < 0.05) is markedly different to control and cadmium group

The average testicular weight was markedly different (*p* < 0.05) across all groups. In contrast to control, average testicular weight decreased markedly (p < 0.05) in cadmium group. The different doses of PREOG averted the cadmium effects on the average testicular weight and the increase was marked (*p* < 0.05) contrary to cadmium group, but the difference was insignificant (*p* > 0.05) to control. However, in group G (1.05 ± 0.17) average testicular weight was insignificant (*p* > 0.05) in contrast to control A (1.35 ± 0.34) and cadmium group B (0.95 ± 0.10). Furthermore, the different doses of PREOG alone significantly increased the average testicular weight (*p* < 0.05) in contrast to cadmium group. However, in contrast to control the average testicular weight was insignificant (*p* > 0.05) (Table 2).

The gonadosomatic index (GSI) was markedly different (*p* < 0.05) across all groups. When contrasted to control, the GSI value of cadmium group decreased markedly (*p* < 0.05). Moreover, the different doses of PREPG averted the cadmium effects, markedly increase the GSI value (*p* < 0.05) contrary to cadmium group, and the increase was marked (*p* < 0.05) in contrast to the control. The GSI level of the different doses of PREOG alone was insignificant (p > 0.05) to control. However, the GSI value was markedly different (*p* < 0.05) to the cadmium-treated group (Table 2).

The epididymal index (EPI) differs markedly (*p* < 0.05) across all groups. In contrast to control, the EPI of cadmium group decreased markedly (*p* < 0.05). The different doses of PREOG abrogated the cadmium effects, markedly increasing the EPI value (*p* < 0.05) contrary to the cadmium group, and the increase was also markedly different (*p* < 0.05) to the control. Furthermore, the EPI value of the different doses of PREOG alone was markedly different (*p* < 0.05) in the control and cadmium groups. However, in groups C (0.06 ± 0.003) there was no marked difference (p > 0.05) contrary to control A (0.06 ± 0.003) (see Table 2).

### Testicular Length and Diameter in Pubertal Male Offspring (PND90)

Table 3 shows testicular length and diameter in pubertal male offspring (PND90). The left and right testicular length was markedly different (*p* < 0.05) across all groups. There was no marked difference (p > 0.05) in the left and right testicular length of cadmium group in contrast to control. The different doses of PREOG averted the cadmium toxic effects, increased the left and right testicular length (*p* < 0.05) markedly in contrast to cadmium group, but the increase was insignificant (*p* > 0.05) to control. However, groups G (18.63 ± 1.93) of the right testicular length difference was insignificant (*p* > 0.05) compared to cadmium group B (17.60 ± 2.30), but in group E (18.96 ± 0.68), left/right testicular length/diameter was markedly different (*p* < 0.05) to control A (17.16 ± 1.60). The left and right testicular length following the treatment with different doses of PREOG alone was insignificant (p > 0.05) to control, although, in group E (15.17 ± 1.31), right testicular length was markedly different (*p* < 0.05) to control A (17.16 ± 1.60). Additionally, the different doses of PREOG alone exerted a marked effect on testicular length (*p* < 0.05) in contrast to cadmium group. However, in groups C and D (17.31 ± 2.20 and 17.31 ± 2.20), right testicular length was not markedly different (*p* > 0.05) to cadmium group B (17.22 ± 1.25), and in group C (1.03 ± 0.36) the left testicular length was also insignificant (p > 0.05) to cadmium group B (0.89 ± 0.07) (Table 3).

**Table 3:** Testicular biometry of pubertal male offspring (PND90) exposed to PREOG/cadmium during gestation and lactation

Diameter/length/ groups	Left testicular length (mm)	Right testicular length (mm)	Left testicular Diameter (mm)	Right testicular diameter (mm)
A	0.99 ± 0.34	17.16 ± 1.60	17.09 ± 1.61	9.36 ± 1.03
B	0.89 ± 0.07	17.22 ± 1.25	17.71 ± 0.60	9.71 ± 0.67
C	1.03 ± 0.36	17.60 ± 2.30	17.47 ± 2.66	9.85 ± 1.17
D	1.10 ± 0.15^b^	17.31 ± 2.20	18.40 ± 0.92	9.70 ± 0.37
E	1.23 ± 0.07^b^	18.96 ± 0.68^ab^	18.9 ± 0.99^ab^	10.79 ± 0.90^ab^
F	1.34 ± 0.26^b^	18.63 ± 1.93	19.02 ± 1.94	10.93 ± 1.26^ab^
*p*	0.0711	0.4268	0.3353	0.0766

Values are express as mean ± SD *n* = 5. *P* < 0.05 using one-way ANOVA is markedly different across all groups. Using student t-test. Superscript ^a b^ = (*p *< 0.05) is markedly different to control and cadmium group

The left testicular diameter was markedly different (*p* > 0.05) across all groups. In contrast to control, the left testicular diameter was also insignificant (p > 0.05) in cadmium group. The left testicular diameter of the different doses of PREOG co-treated with cadmium was markedly different (*p* < 0.05) to cadmium and control group. However, group G (19.02 ± 1.94), left testicular diameter was insignificant (p > 0.05) to control A (17.09 ± 1.61), and group G (19.02 ± 1.94) left testicular diameter was also insignificant (*p* > 0.05) to cadmium group B (17.71 ± 0.60). Furthermore, the different doses of PREOG alone did not exert marked effects on the left testicular diameter when contrasted to control and cadmium group (Table 3).

The right testicular diameter was markedly different (*p* < 0.05) across all groups. In contrast to control, the right testicular diameter was insignificant (*p* > 0.05) in cadmium group. The different doses of PREOG averted the cadmium effect, and increased the right testicular diameter (*p* < 0.05) markedly contrary to the cadmium group, and the increase was also markedly different (*p* < 0.05) from to control. Furthermore, the right testicular diameter was insignificant (p > 0.05) to the control and cadmium group following the treatment with the different doses of PREOG alone (Table 3).

### Semen Quality in Pubertal Male Offspring (PND90)

Table 4. Shows the semen quality of pubertal male offspring (PND90) exposed to cadmium and PREOG. The sperm motility was markedly different (*p* < 0.05) across all groups. In contrast to control, there was a marked decrease in sperm motility (*p* < 0.05) in cadmium group. The different doses of PREOG abrogated the cadmium effects, and enhanced the sperm motility (*p* < 0.05) significantly in contrast to cadmium group, and the increase was also markedly different (*p* < 0.05) from the control. Sperm motility was insignificant (p > 0.05) to control following the treatment with different doses of PREOG alone. However, when contrasted with cadmium group there was a marked difference (*p* < 0.05) (Table 4).

**Table 4:** The semen quality of pubertal male offspring (PND90) exposed PREOG/cadmium during gestation and lactation

Semen analysis & morphology/ groups	Sperm motility (%)	Sperm livability (%)	Sperm count (X106 sperm/mls)	Head defect (%)	Middle defect (%)	Tail defect (%)	Total morphological abnormality (%)
A	81.8 ± 8.87	83.0 ± 5.70	112.8 ± 9.36	1.19 ± 0.52	5.09 ± 0.99	4.73 ± 0.42	11.01 ± 1.93
B	40.0 ± 2.31^a^	43.0 ± 9.75^a^	69.6 ± 5.13^a^	2.01 ± 0.54^**a**^	9.34 ± 0.21^**a**^	16.01 ± 0.01^**a**^	27.37 ± 0.76^**a**^
C	87.4 ± 4.88^b^	78.0 ± 8.37^b^	106.2 ± 4.76^b^	1.06 ± 0.11^**b**^	5.02 ± 0.35^**b**^	6.71 ± 1.98^**ab**^	12.79 ± 2.44^**b**^
D	86.8 ± 13.22^b^	79.6 ± 8.85^b^	106.4 ± 6.88^b^	0.73 ± 0.98^**b**^	7.01 ± 0.22^**ab**^	5.03 ± 1.09^**b**^	12.77 ± 2.29^**b**^
E	69.2 ± 9.78^ab^	68.4 ± 4.16^ab^	87.8 ± 7.79^ab^	1.98 ± 0.71a	7.69 ± 1.97^**ab**^	6.90 ± 2.33^**ab**^	16.57 ± 5.01^ab^
F	66.0 ± 9.62^ab^	74.6 ± 12.97^b^	95.0 ± 6.32^ab^	1.17 ± 0.45^**b**^	7.19 ± 1.63^**ab**^	6.01 ± 0.12^ab^	14.37 ± 2.20^**ab**^
*p*	0.0001	0.0001	0.0001	0.0001	0.0001	0.0001	0.0001

Values are express as mean ± SD (*n* = 5). *P* < 0.05 using one-way ANOVA is markedly different across all groups. Using student t-test. Superscript ^a b^ = (*p* < 0.05) is markedly different to control and cadmium group

The sperm livability also was markedly different (*p* < 0.05) across all groups. In contrast to control group, sperm livability decreased markedly (*p* < 0.05) in cadmium group. The different doses of PREOG mitigated the cadmium effects, significantly increasing sperm livability (*p* < 0.05) contrary to the cadmium group, but the increase was insignificant (*p* > 0.05) to control. However, in group E (68.4 ± 4.16), sperm livability differs markedly (*p* < 0.05) to control A (83.0 ± 5.70). Furthermore, sperm livability was insignificant (p > 0.05) to control after treatment with the different doses of PREOG alone. However, there was a marked difference (*p* < 0.05) when contrasted to the cadmium group (Table 4).

The sperm count was markedly different (*p* < 0.05) across all groups. In contrast to control group, sperm count decreased markedly (*p* < 0.05) in the cadmium group. The different doses of PREOG abrogated the cadmium effects, markedly increasing the sperm count (*p* < 0.05) contrary to the cadmium group, and the increase was also markedly different (*p* < 0.05) to control. Furthermore, the sperm count was insignificant (p > 0.05) to the control after treatment with the different doses of PREOG alone. However, there was a marked difference (*p* < 0.05) when contrasted to the cadmium group (Table 4).

The total morphological abnormality was evaluated by the following defects: head defects, middle piece defects, and tail defects. The Head defects in this study differ markedly (*p* < 0.05) across all groups. In contrast to the control, the head defects increase markedly (*p* < 0.05) in the cadmium group. The different doses of PREOOG abrogated the cadmium effects, markedly decreasing the head defects (*p* < 0.05) contrary to the cadmium group, but the decrease was insignificant (*p* > 0.05) to control. Furthermore, the head defect was insignificant (p > 0.05) to the control after treatment with the different doses of PREOG. However, there was a marked difference (*p* < 0.05) when contrasted to the cadmium group (Table 4).

The Middle defects also showed marked differences (*p* < 0.05) across all groups. When compared to the control, there was a marked increase in the middle defects (*p* < 0.05) of the cadmium group. The different doses of PREOG averted the cadmium effects, and markedly decreased the middle defects (*p* < 0.05) in contrast to the cadmium group, and the decrease was also markedly different (*p* < 0.05) to control. Furthermore, the middle defect decreased markedly (*p* < 0.05) by treatment with the different doses of PREOG alone in contrast to the control and cadmium group. However, in group C (5.02 ± 0.35) the middle defect was insignificant (p > 0.05) to control A (5.09 ± 0.99) (Table 4).

The Tail defect differs markedly (*p* < 0.05) across all groups. In contrast to the control, tail defect increased notably (*p* < 0.05) in cadmium group. The different doses of PREOG averted the cadmium effects, and markedly decreased the tail defects (*p* < 0.05) contrary to cadmium group, and the decrease also was significant (*p* < 0.05) to control. Additionally, the tail defect was markedly different (*p* < 0.05) to control and cadmium group by the treatment with different doses of PREOG alone. However, in groups D (5.03 ± 1.09**)** tail defect was insignificant (*p* > 0.05) to control A (4.73 ± 0.42) (Table 4).

The Total percentage of morphological abnormality also differs markedly (*p* < 0.05) across all groups. In contrast to control, morphological abnormality increased markedly (*p* < 0.05) in cadmium group. The different doses of PREOG were able to abrogate the cadmium effects, and significantly decrease the total morphological abnormality (*p* < 0.05) contrary to the cadmium group, and the decrease was also markedly different (*p* < 0.05) to control. The total percentage of morphological abnormality was markedly decreased (*p* < 0.05) compared to cadmium group by the different doses of PREOG. However, there was no significant difference (*p* > 0.05) when contrary to control (Table 4).

### Serum Hormonal Level in Pubertal Male Offspring (PND90)

**Fig. 1:**
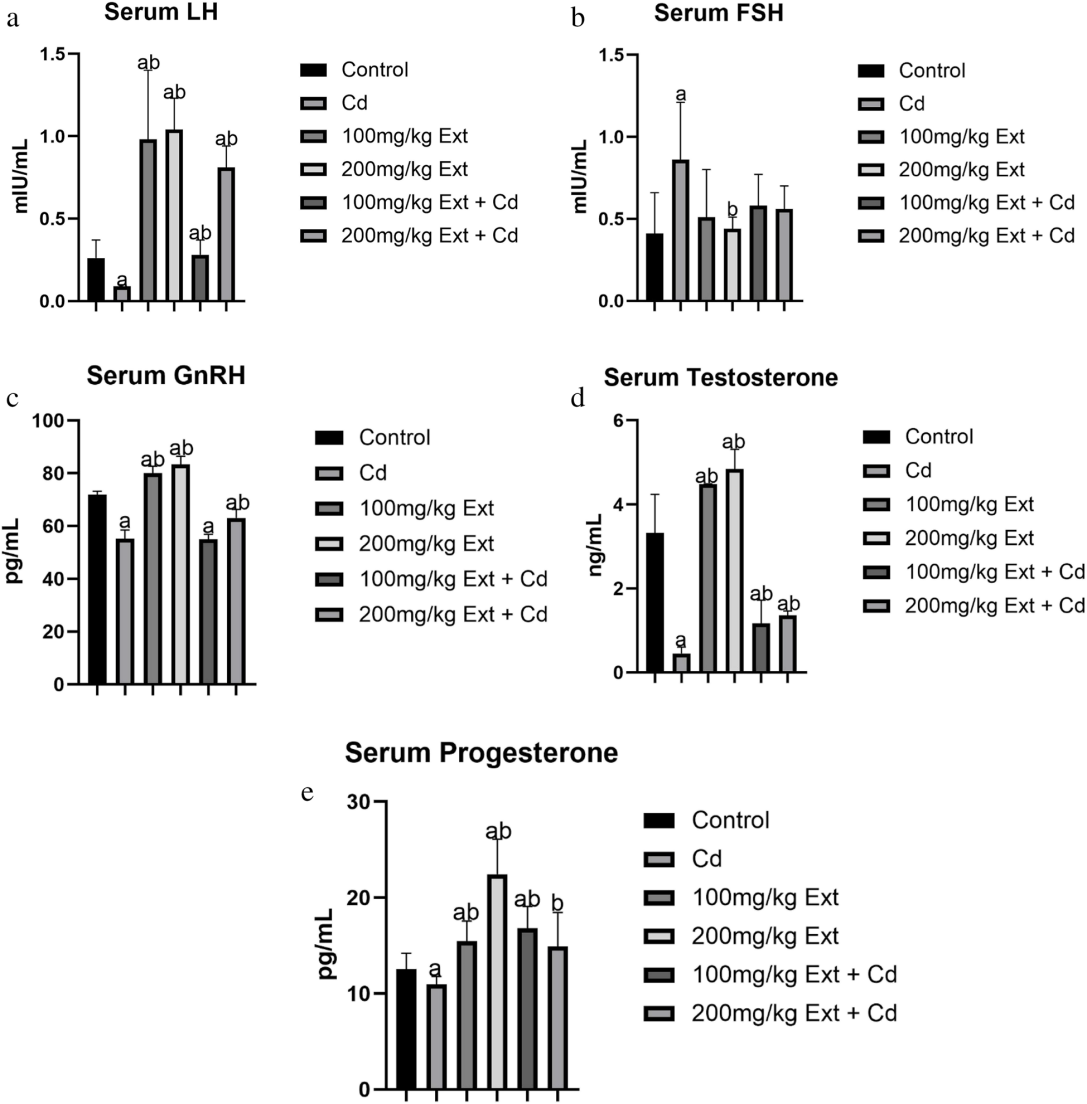
**a**. Serum LH level in PND 90 offspring treated with Cd and PREOG. Values expressed in mean ± SD. Superscripts (**a** & **b**) indicate a notable difference from the control and cadmium groups. Abbreviations: Cd; CdCL_2_, Ext; PREOG, LH; luteinizing hormone. **b**. Serum FSH level in PND 90 offspring treated with Cd and PREOG. Values expressed in mean ± SD. Superscripts (**a** & **b**) indicate a notable difference from the control and cadmium groups. Abbreviations: Cd; CdCL_2_, Ext; PREOG, FSH; follicle stimulating hormone. **c**. Serum GnRH level in PND 90 offspring treated with Cd and PREOG. Values expressed in mean ± SD. Superscripts (**a** & **b**) indicate a notable difference from the control and cadmium groups. Abbreviations: Cd; CdCL_2_, Ext; PREOG, GnRH; gonadotropin-releasing hormone. **d**. Serum testosterone level in PND 90 offspring treated with Cd and PREOG. Values expressed in mean ± SD. Superscripts (**a** & **b**) indicate a notable difference from the control and cadmium groups. Abbreviations: Cd; CdCL_2_, Ext; PREOG. **e**. Serum progesterone level in PND 90 offspring treated with Cd and PREOG. Values expressed in mean ± SD. Superscripts (**a** & **b**) indicate a notable difference from the control and cadmium groups. Abbreviations: Cd; CdCL_2_, Ext; PREOG

Figure 1a shows the hormonal profile in pubertal male offspring (PND90) exposed to cadmium, PREOG during gestation and lactation. At PND 90, there was a marked difference (*p* < 0.05) in serum LH value across all groups. In contrast to control, there was a marked decrease of serum LH value (*p* < 0.05) in cadmium group. The different doses of PREOG mitigated the cadmium effects, markedly increased serum LH value (*p* < 0.05) contrary to the cadmium group, and the increase also was statistically significant (*p* < 0.05) to control. However, in the group treated with 100 mg/kg PREOG + CdCL_2_, the LH value was insignificant (p > 0.05) to control. Furthermore, serum LH value after treatment with the different doses of PREOG alone was markedly different (*p* < 0.05) when contrasted to control and cadmium groups (Fig. 1a)

Serum FSH value was insignificant (p > 0.05) across all groups. In contrast to control, there was a marked increase in serum FSH value (*p* < 0.05) in cadmium group. The different doses of PREOG averted the cadmium effects, and decreased serum FSH level (*p* < 0.05) markedly contrary to the cadmium group, although, in the group treated with 100 mg/kg PREOG + CdCL_2_, FSH value was insignificant (p > 0.05) to cadmium group, in contrast to control, there was no marked difference (p > 0.05) in serum FSH level. Furthermore, the effects of different doses of PREOG alone on serum FSH levels were insignificant (*p* > 0.05) when contrasted to the control and cadmium groups (Fig. 1b).

Serum testosterone value was markedly different (*p* < 0.05) across all groups. In contrast to the control, there was a marked decrease in serum testosterone value (*p* < 0.05) in the cadmium group. The different doses of PREOG abrogated the cadmium effects, increased serum testosterone value (*p* < 0.05) significantly contrary to the cadmium group, and the increase also differs markedly (*p* < 0.05) to control. Furthermore, serum testosterone value by the treatment with different doses of PREOG alone differs markedly (*p* < 0.05) when contrasted to control and cadmium groups (Fig. 1c)

Serum progesterone value was different markedly (*p* < 0.05) across all groups. In contrast to control group, serum progesterone value decreased significantly (*p* < 0.05) in cadmium group. The different doses of PREOG mitigated the cadmium effects, increased serum progesterone value (*p* < 0.05) markedly contrary to the cadmium group, but the increase was insignificant (p > 0.05) when contrasted to the control, but the group treated with 100 mg/kg PREOG + CdCL_2_, serum progesterone value was markedly different (*p* < 0.05) to control. Furthermore, the effects of the different doses of PREOG alone on serum progesterone value differs markedly (*p* < 0.05) contrary to control and cadmium group (Fig. 1d).

The serum GnRH value difference was also marked (*p* < 0.05) across all groups. In contrast to control, there was a marked decrease in serum GnRH level (*p* < 0.05) in cadmium group. Co-treatment with different doses of PREOG and cadmium, abrogated the cadmium effect and increased serum GnRH value (*p* < 0.05) markedly contrary to the cadmium group, and the increase was also marked (*p* < 0.05) to control. However, in the group treated with 200 mg/kg PREOG + CdCL_2_, the GnRH value was insignificant (p > 0.05) when contrasted to the cadmium group. Additionally, the effects of the different doses of PREOG treatment alone on serum GnRH level differ markedly (*p* < 0.05) when contrasted to control and cadmium groups (Fig. 1e).

### 3β-HSD, 17β-HSD and StAR Steroidogenic Genes in Pubertal Male Offspring (PND90)

**Fig. 2:**
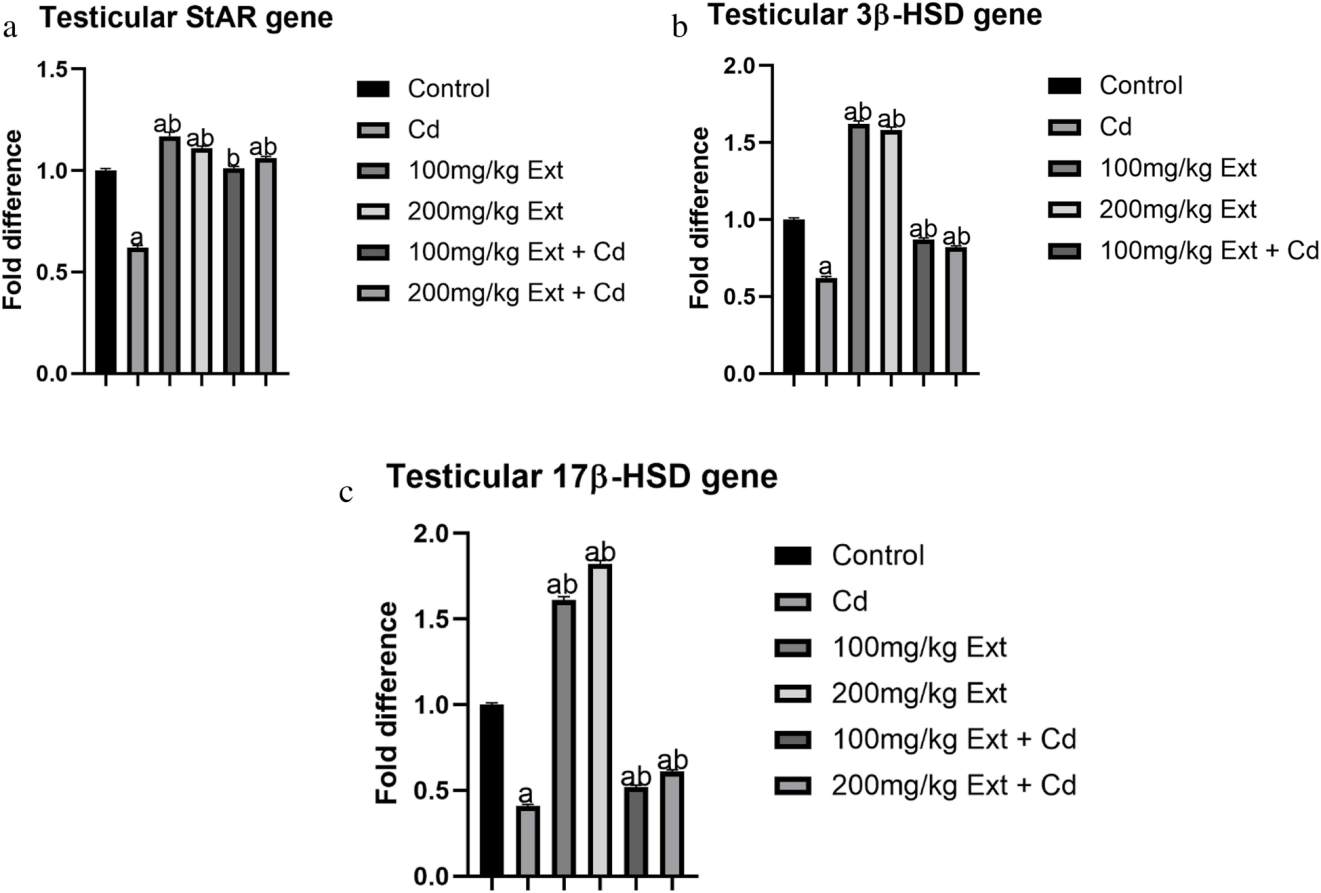
**a**. Testicular StAR gene expression in PND 90 offspring treated with Cd and PREOG. Values expressed in mean ± SD. Superscripts (**a** & **b**) indicate a notable difference from the control and cadmium groups. Abbreviations: Cd; CdCL_2_, Ext; PREOG, StAR; steroidogenic acute regulatory proteins. **b**. Testicular 3β-HSD gene expression in PND 90 offspring treated with Cd and PREOG. Values expressed in mean ± SD. Superscripts (**a** & **b**) indicate a notable difference from the control and cadmium groups. Abbreviations: Cd; CdCL_2_, Ext; PREOG, 3β-HSD; beta-hydroxysteroid dehydrogenase. **c**. Testicular 17β-HSD gene expression in PND 90 offspring treated with Cd and PREOG. Values expressed in mean ± SD. Superscripts (**a** & **b**) indicate a notable difference from the control and cadmium groups. Abbreviations: Cd; CdCL_2_, Ext; PREOG, 17β-HSD; beta-hydroxysteroid dehydrogenase

Figures 2a, b, and c for the pubertal evaluation of testicular steroidogenesis following treating dam with PREOG and cadmium during gestation and lactation. The values 3β-HSD, 17β-HSD, and StAR genes were assessed at (PND90) using qRT-PCR (Figs. 2a-c). From our result, the treatment with cadmium significantly downregulates the steroidogenic genes 3β-HSD, 17β-HSD, and StAR markedly (*p* < 0.05) in contrast to control. However, the co-treatment with the different doses of PREOG abrogated the cadmium effects, significantly upregulating the steroidogenic genes (3β-HSD, 17β-HSD, and StAR) (*p* < 0.05) in contrast to the cadmium group, and the increase was significant (*p* < 0.05) in contrast to the control group. However, there was no marked difference (*p* > 0.05) of the StAR gene in the 100 mg/kg plus 3 mg/kg CdCL_2_ treated groups when contrasted to the control. Additionally, the different doses of PREOG upregulated 3β-HSD, 17β-HSD, and StAR steroidogenic genes markedly (*p* < 0.05) contrary to the control and cadmium group (Figs. 2a-c).

### Testicular PTMA Gene Expression in Pubertal Male Offspring (PND90)

**Fig. 3:**
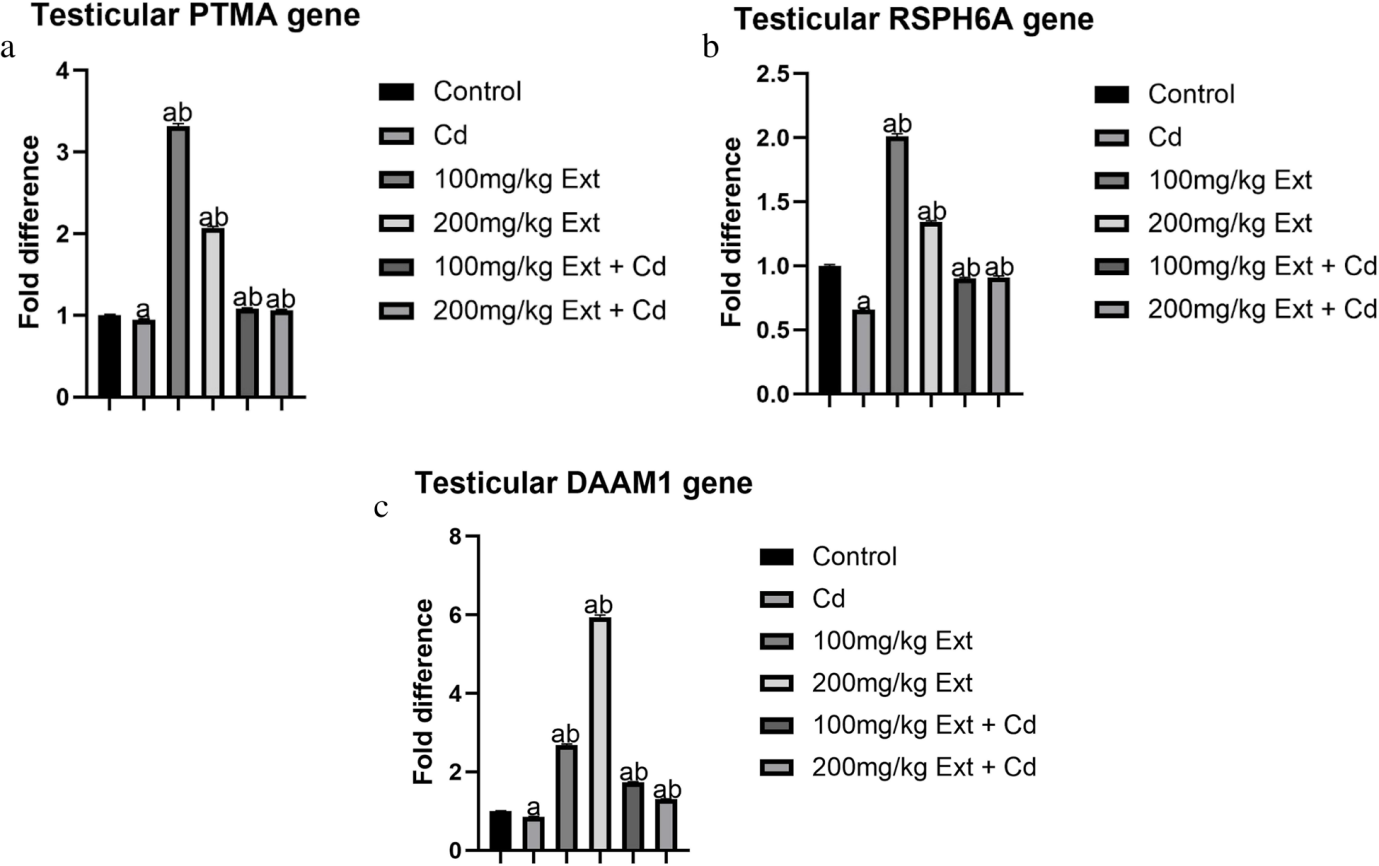
**a **Testicular PTMA gene expression in PND 90 offspring treated with Cd and PREOG. Values expressed in mean ± SD. Superscripts (**a** & **b**) indicate a notable difference from the control and cadmium groups. Abbreviations: Cd; CdCL_2_, Ext; PREOG, PTMA; prothymosin alpha. **b** Testicular RSPH6A gene expression in PND 90 offspring treated with Cd and PREOG. Values expressed in mean ± SD. Superscripts (**a** & **b**) indicate a notable difference from the control and cadmium groups. Abbreviations: Cd; CdCL , Ext; PREOG, RSPH6A; Radial spoke head 6 homolog A. **c** Testicular DAAM 1 gene expression in PND 90 offspring treated with Cd and PREOG. Values expressed in mean ± SD. Superscripts (**a** & **b**) indicate a notable difference from the control and cadmium groups. Abbreviations: Cd; CdCL_2_, Ext; PREOG, DAAM 1; Disheveled-associated activator of morphogenesis 1

Figure 3a shows testicular PTMA gene in pubertal male offspring (PND90) exposed to cadmium, and PREOG during gestation and lactation. To evaluate testicular capacity to produce spermatozoa with intact acrosome integrity necessary for fertilization, using quantitative real-time polymerase chain reaction (qRT-PCR) testicular prothymosin α (PTMA) genes were assayed. From our result, treatment with cadmium significantly downregulates the PTMA gene (*p* < 0.05) contrary to the control. However, the different doses of PREOG mitigate the cadmium effect, and markedly upregulate PTMA gene level, and the increase was also markedly (*p* < 0.05) to the control group. Furthermore, the different doses of PREOG alone upregulated the PTMA value markedly (*p* < 0.05) when contrasted with the control and cadmium groups (see Fig. 3a)

### Testicular RSPH6A Gene Expression in Pubertal Male Offspring (PND90)

Figure 3b shows testicular RSPH6A gene in pubertal male offspring (PND90) exposed to cadmium/PREOG during gestation and lactation. To evaluate pubertal testicular flagellate motility potential which is needed for the motility of matured spermatozoa, the radial spoke head 6 homolog A (RSPH6A) gene was assayed with qRT-PCR. From our result, cadmium treatment markedly downregulates the RSPH6A gene level (*p* < 0.05) contrary to the control group. The different doses of PREOG mitigate the cadmium effects and markedly upregulate the RSPH6A gene level (*p* < 0.05) in contrast to the cadmium group, and the increase also was statistically significant (*p* < 0.05) when contrasted to the control group. Furthermore, the different doses of PREOG alone upregulate the RSPH6A gene level markedly (*p* < 0.05) in contrast to the control and cadmium group (Fig. 3b)

### Testicular DAAM1 Gene Expression in Pubertal Male Offspring (PND90)

To evaluate pubertal testicular cytoskeletal architecture potential which is translated to structural integrity of matured spermatozoa, the disheveled-associated activator of morphogenesis 1 (DAAM1) gene was assayed for with q-RT-PCR on PND90 following exposure to cadmium, PREOG during gestation and lactation (Fig. 3c). From our result, cadmium treatment markedly downregulates the DAMI gene level (*p* < 0.05) in contrast to the control group. The different doses of PREOG mitigate the cadmium effect and markedly upregulate the DAMI gene level (*p* < 0.05) in contrast to the cadmium group, and the increase also was markedly different *p* < 0.05 in contrast to control. Furthermore. the different doses of PREOG alone upregulate the DAMI gene level markedly (*p* < 0.05) in contrast to the control and cadmium group (Fig. 3c)

### Testicular Oxidative Status in Pubertal Male Offspring (PND90)

Table 5 shows testicular oxidative index in pubertal male offspring (PND90) exposed to cadmium, PREOG during gestation and lactation. At (PND90) the hydrogen peroxide (H_2_O_2_) value was insignificant (*p* > 0.05) across all groups.in contrast to the control, the H_2_O_2_ value increased markedly (*p* < 0.05) in the cadmium group. Co-treatment with PREOG and cadmium, H_2_O_2_ values were comparable to cadmium and control group, but in group F (30.41 ± 6.72) H_2_O_2_ value differed markedly (*p* < 0.05) in the cadmium group B (37.50 ± 5.24). In the treatment with different doses of PREOG alone, the H_2_O_2_ value was insignificant (p > 0.05) to control. However, there was a marked difference (*p* < 0.05) when contrasted to the cadmium group (Table 5).

**Table 5:** The oxidative status of prepubertal male offspring (PND 90) exposed to PREOG/Cd during gestation and lacation

Groups	H_2_O_2_ (nmol/g protein)	GSH (mg/ g protein)	MDA (nmol/mg protein)	GPX (U/mg protein)	GST (U/mg protein)	SOD (U/mg protein)
A	28.56 ± 3.78	71.66 ± 10.46	2.03 ± 0.38	72.71 ± 2.44	7.10 ± 3.48	79.81 ± 24.55
B	37.50 ± 5.24^a^	47.23 ± 2.31^a^	3.20 ± 0.89^a^	68.41 ± 1.36^a^	1.87 ± 1.04^a^	34.03 ± 4.67^a^
C	28.66 ± 5.92^b^	68.93 ± 8.30^b^	1.78 ± 0.21^b^	71.27 ± 4.96	4.90 ± 2.08^b^	64.23 ± 7.37^b^
D	26.48 ± 2.29^b^	71.77 ± 6.09^b^	1.92 ± 0.20^b^	75.35 ± 6.52^b^	7.14 ± 3.16^b^	79.81 ± 4.97^b^
E	32.37 ± 7.25	52.19 ± 3.98^ab^	2.56 ± 1.07	82.08 ± 4.55^ab^	2.75 ± 1.52^a^	43.40 ± 12.27^ab^
F	30.41 ± 6.72^b^	54.43 ± 1.93^ab^	2.56 ± 1.28	80.59 ± 7.06^ab^	3.72 ± 1.88^ab^	41.67 ± 9.82^ab^
*p*	0.0561	0.0001	0.0827	0.0001	0.0055	0.0001

Values are express as mean ± SD *n* = 5. *p* < 0.05 using one-way ANOVA is markedly different across all groups. Using student t-test. ^a b^ *p* < 0.05 is markedly different to control and cadmium group

Abbreviations: *Cd* CdCL2,* GPx* glutathione peroxidase, *GST* glutathione S-transferase, *SOD* superoxide dismutase, *H2O2* hydrogen peroxide, *GSH* reduced glutathione, *MDA* malondialdehyde

Reduced glutathione (GSH) value was markedly different (*p* < 0.05) across all groups. In contrast to the control, the GSH value (*p* < 0.05) decreased markedly in the cadmium group. The different doses of PREOG averted the cadmium effects, and increased the GSH value (*p* < 0.05) markedly compared to cadmium group, and the increase was also markedly different (*p* < 0.05) to the control. Furthermore, the effects of different doses of PREOG alone on GSH value was insignificant (*p* > 0.05) to control, but when contrasted to cadmium group, GSH value differs markedly (*p* < 0.05) (Table 5).

Lipid peroxidation was evaluated by the formation of thiobarbituric acid reacting substances (TBARS) from malondialdehyde (MDA). As indicated in Table 5 the MDA value was markedly different (*p* < 0.05) across all groups. In contrast to control, the MDA value (*p* < 0.05) increased markedly in cadmium group. Co-treatment of different doses of PREOG with cadmium, MDA values are comparable to cadmium group and control. Furthermore, the effects of different doses of PREOG alone treatment on MDA value was insignificant (p > 0.05) to control, but MDA value differs (*p* < 0.05) when contrasted to cadmium group (Table 5).

Glutathione peroxidase activity (GPx) activity differs markedly (*p* < 0.05) across all groups. In contrast to control, the GPx value (*p* < 0.05) reduced markedly in the cadmium group. The cadmium effect was mitigated by co-treatment with different doses of PREOG, and the increase GPx value was markedly different (*p* < 0.05) from to the cadmium group, and the increase was also markedly different (*p* < 0.05) to control. Additionally, the different doses of PREOG alone on GPx values were insignificant (*p* > 0.05) to the control and cadmium group. However, the GPx value in group D (75.35 ± 6.52) differs markedly (*p* < 0.05) from cadmium group B (68.41 ± 1.36) (Table 5).

Glutathione S-transferase (GST) activity was markedly different (*p* < 0.05) across all groups. In contrast to the control, GST value decreased markedly (*p* < 0.05) in the cadmium group. Co-treatment of PREOG with cadmium, GST value was comparable to the cadmium group, but differs markedly (*p* < 0.05) from the control. However, in group F (3.72 ± 1.88) GST value differs markedly from cadmium group B (1.87 ± 1.04). Additionally, the effects of different doses of PREOG alone on GST value were insignificant (p > 0.05) to control, but were markedly different (*p* < 0.05) when contrasted to cadmium group (Table 5).

Superoxide dismutase (SOD) activity was also markedly different (*p* < 0.05) across all groups. In contrast to control, the SOD value (*p* < 0.05) decreased markedly in the cadmium group. The cadmium effect was mitigated by co-treatment of the different doses of PREOG, the SOD value increased markedly (*p* < 0.05) contrary to the cadmium group, and the increase was also markedly different (*p* < 0.05) to the control. Additionally, the effect of different doses of PREOG alone on the SOD activity was insignificant (p > 0.05) to control, but there was a marked difference (*p* < 0.05) when contrasted to the cadmium group (Table 5).

### Fertility Index in Pubertal Male Offspring (PND90)

Table 6 shows the fertility index in pubertal male offspring (PND90) exposed to cadmium, PREOG during gestation and lactation. In this study the fertility index was evaluated using: the male and female fertility index, gestation and parturition index, mean percentage of pups delivered alive, the pups birth weight, and the survival index for the first seven days after parturition. Male offspring (PND90) of dam exposed to cadmium during gestation and lactation had a 50% decrease of their fertility index in contrast to control, there was also a 50% decrease in female fertility index, the gestation/lactation index was equally decreased by 50% in contrast to control (Table 6). The different doses of PREOG averted the cadmium effect and enhanced the male/female fertility index and gestation/parturition index by 25% in contrast to the cadmium group, but the increase was not comparable to the control. Furthermore, the male/female fertility index and gestation/parturition index of the different doses of PREOG alone were comparable to the control. However, there was a 50% increase in the male/female fertility index and gestation/lactation index contrary to the cadmium group (Table 6).

**Table 6:** The fertility index of pubertal male offspring (PND90) exposed to PREOG/cadmium during gestation and lactation

Groups	Male fertility Index (%)	Female fertility Index (%)	Parturition Index (%)	Gestation Index (%)	Mean ± SD of delivered pups (%)	Mean ± SD Birth weight Delivered (g)	Survival Index from PND1 to PND7 (%)
A	100	100	100	100	8.9 ± 0.3	5.49 ± 0.49	88.00 ± 4.47
B	50	50	50	50	5.9 ± 0.2a	4.05 ± 0.41a	86.00 ± 5.47
C	100	100	100	100	8.1 ± 0.1ab	5.24 ± 0.17b	86.00 ± 5.48
D	100	100	100	100	8.5 ± 3.2b	5.55 ± 0.26b	84.00 ± 5.48
E	80	80	80	80	8.0 ± 0.1ab	4.87 ± 0.13ab	86.00 ± 5.50
F	100	100	100	100	7.4 ± 0.5ab	5.31 ± 0.11b	84.00 ± 5.46
*p*					0.0001	0.0001	0.8440

Values are express as mean ± SD *n* = 5. *P* < 0.05 using one-way ANOVA is markedly different across all groups. Using student t-test. Superscript ^a b^ = (*p* < 0.05) is markedly different to control and cadmium group

The mean percentage of pups delivered was markedly different (*p* < 0.05) across all groups. In contrast to control, the mean % of pups delivered (*p* < 0.05) decreased markedly in cadmium group. The different doses of PREOG averted the cadmium effects, increased the mean % of pups delivered (*p* < 0.05) markedly contrary to the cadmium group, and the increase was also markedly different (*p* < 0.05) from the control. Additionally, the different doses of PREOG alone increased the mean % of pups delivered markedly (*p* < 0.05) contrary to the cadmium group, although there was also an insignificant difference (*p* > 0.05) when in contrast to the control. However, in groups C (8.1 ± 0.1) mean % of pups delivered differs markedly (*p* < 0.05) to control A (8.9 ± 0.3) (Table 6).

The mean birth weight difference was marked (*p* < 0.05) across all groups. In contrast to control, the mean birth weight (*p* < 0.05) decreased markedly in cadmium group. The different doses of PREOG abrogated the cadmium effects, increasing the mean birth weight (*p* < 0.05) markedly in contrast to the cadmium group, but the increase was insignificant (*p* > 0.05) to control. Moreover, the mean birth weight was not markedly different (*p* > 0.05) to control of the groups treated with different doses of PREOG alone. However, there was a marked difference (*p* < 0.05) when in contrast to the cadmium group (see Table 6).

The survival index from postnatal day one to postnatal day seven was insignificant (p > 0.05) across all groups. When in contrast to the control, the survival index of the cadmium group was insignificant (*p* > 0.05). Co-treatment of different doses of PREOG with cadmium, the survival index was comparable (p > 0.05) to cadmium and control group. Furthermore, the treatment with different doses of PREOG alone did not show any marked difference in survival index (*p* > 0.05) when contrasted to the control and cadmium group (Table 6).

#### The Histopathology of the Testes in Pubertal Male Offspring (PND90)

**Fig. 4:**
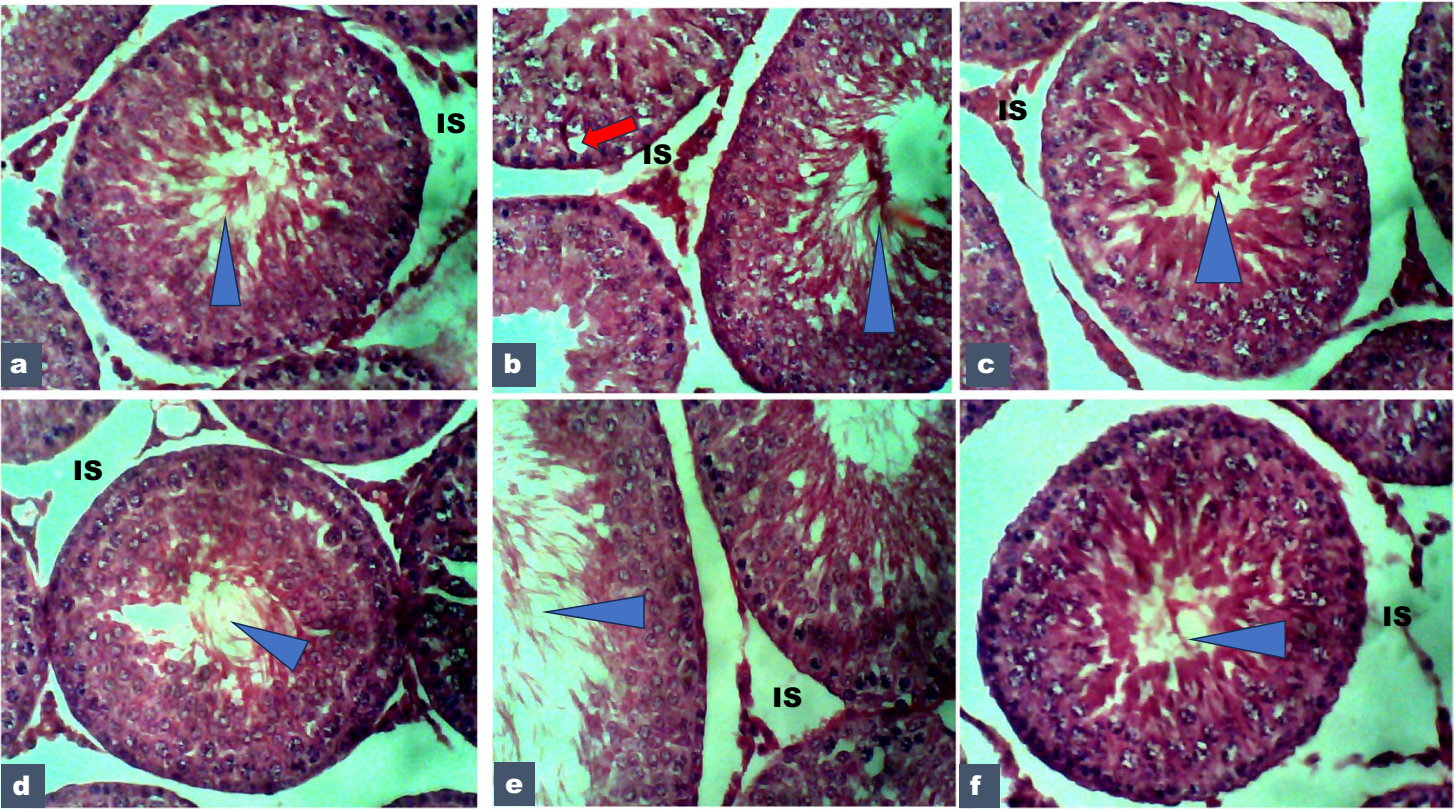
The photomicrograph of the testicular tissues in PND 90 offspring stained with H/E (X 400) **a** (control), **b** (3mk/kg CdCL), **c** (100 mg/kg PREOG), **d** (200 mg/kg PREOG), **e** (100 mg/kg PREOG + 3 mg/kg CdCL_2_) and **f** (200 mg/kg PREOG + 3 mg/kg CdCL_2_)

Figure 4 shows the histopathology of the testes in pubertal male offspring (PND90) exposed to cadmium and PREOG during gestation and lactation. To evaluate the histological characteristics of the testis in pubertal male offspring (PND90) of dam exposed to cadmium and PREOG Hematoxylin–eosin staining was performed (Fig. 4). The control, PREOG alone treated rats presented an intact seminiferous epithelium and germinal cells in all the different stages of differentiation and with lumens filled with mature spermatozoa (blue triangle). The interstitium is with intact blood vessels and Leydig cells (**IS**). Exposure to cadmium altered the homogenous testicular structure (Fig. 4) when compared to the control, characterized by intracellular vacuolation (red arrow) close to the developing spermatogonia, the intercellular space hemorrhage. However, co-treatment of the dam with PREOG and alpha-tocopherol mitigated the cadmium-induced testicular irregularity and restored/improved the normal morphological structure of the testes with active spermatogenesis in seminiferous tubules (Fig. 4).

## Discussion

In our previous study, we reported that the exposure of dam to cadmium during gestation and lactation impaired reproductive potential in prepubertal male offspring (PND30), and that co-treatment with PREOG abrogated the cadmium effect and enhanced the reproductive potential. This is a continuation of the study, and it is to ascertain if the enhanced reproductive potential in prepubertal male offspring (PND30) by PREOG, translates to improved reproductive outcomes at the pubertal or adult age of the male offspring (PND90). Our focus was on testicular biometry (gonadosomatic and epididymal index, left and right testicular diameter/length, histology/TEM of the testes), semen analysis, and serum hormonal assay. Moreover, testicular expression of StAR, 3β-HSD1 and 17β-HSD3 steroidogenic genes, RSPH6A gene associated with sperm motility, DAAM1 gene associated with the cytoskeleton of the sperm, PTMA gene associated with maintaining normal acrosome integrity and finally fertility study of the pubertal male (PND90) can sire an offspring.

In this study, the body weight gains in the pubertal male offspring (PND90) of the dam treated with cadmium during gestation and lactation were insignificant, even though weight gain across all groups was significant. A similar report of insignificant weight difference has been documented [54]. For the first time in this study, gonadosomatic index (GSI) and epididymis index (EPI) were evaluated in pubertal male offspring (PND90) and our result indicated a significant decrease of GSI and EPI in the cadmium group compared to the control. The closest to this finding was documented by [25] who reported a significant reduction of relative reproductive organs weight of testes, seminal vesicle, and epididymis in the cadmium-exposed group. Additionally, decreased GSI values in adult rats were have also been documented [55, 56]. However, co-treatment with PREOG averted the cadmium-induced GSI and EPI decrease at (PND 90), thus enhancing the reproductive potential in pubertal male offspring (PND90), this attuned with the earlier report elsewhere [41]. Also in this study, left and right testicular length/diameter was insignificant in pubertal male offspring (PND90) in the cadmium group. This finding is contrary to what was documented by [56] who reported decreased left/right testicular length/diameter in adult male rats. This contradiction could be associated with the dosage, route, age, and experimental protocol [52].

Oxidative–mediated toxicity has been implicated as the major route, among the numerous ones that are complicit in the pathology of cadmium-induced testicular insult. Various reports have been documented to assert the complicity of oxidative stress in testicular insult due to cadmium treatment [16]. In this study, dam exposed to cadmium during gestation and lactation induced oxidative stress in the testes of male offspring (PND90), by upregulating oxidative stress markers such as malondialdehyde (MDA) (increased lipid peroxidation rate) and hydrogen peroxide (H_2_O_2)_ values. While at the same time downregulating testicular antioxidant enzymes such as superoxide dismutase (SOD) and Glutathione S-transferase (GST). There was also a significant downregulation of testicular glutathione peroxides (GPx) value. Similar findings of cadmium downregulating testicular antioxidant enzymes and GPx value, while at the same upregulating oxidative stress markers have been documented in adult male rats [57–59]. To our knowledge, this is the first report on cadmium-induced oxidative stress in male offspring (PND90) exposed to cadmium during gestation and lactation. Also in this study, testicular glutathione (GSH) level was downregulated significantly in male offspring (PND90). Similar to what was recorded for adult male rats treated with cadmium by [60, 61]. However, the co-treatment of the dam with PREOG abrogated the cadmium effects by significantly downregulating the oxidative stress markers, while significantly upregulating testicular antioxidant enzymes and GPx value at the same time.

Globally, spermatozoa of adequately high standard (motility, concentration, DNA integrity, and morphology) is the pillar of male fertility [62], and the alarming universal decline of male fertility rate corresponds directly to enhanced impediment of gamete quality being witnessed [62]. The quality of a spermatozoa predisposes it to the capacity to fertilize an oocyte, while its nature prone it to insults that could arise from biological, physical, and chemical intoxicants [63]. In this study, sperm motility, sperm livability, and sperm count were significantly decreased in pubertal male offspring at (PND90) in the cadmium group compared to the control. Findings of reduced sperm production and epididymal spermatogenic parameters in adult male rats upon exposure to cadmium during gestation and lactation were also documented [25, 54]. Furthermore, there was an overall increase in total morphological abnormality which corresponded with increases seen in the head, middle, and tail abnormality of the spermatozoa of adult male rats (PND 90) exposed to cadmium during gestation and lactation. The elevated total morphological abnormality of the head, middle piece, and tail reported in this study could be associated with DNA, protein, or lipid damage of germ cells during gestation and lactation by cadmium treatment of dam [60]. Nevertheless, the reduced semen quality and enhanced semen abnormality of adult males (PND90) were significantly abrogated by co-treating of dam with PREOG during gestation and lactation.

One vital element of spermatogenesis is the energetic cell junction restructuring. This cellular process is anchored by cytoskeletons such as actin, intermediate filament, and microtubule. DAAM1 organizes actin by nucleation, elongation, and probably, bundling actin [64]. In our study, the DAAM1 gene was assayed in the testes of pubertal males (PND90) exposed to cadmium during gestation and lactation and the gene expression was significantly downregulated by the cadmium treatment. A similar finding of inhibited DAAM1 proteins was documented by [25], and impaired testicular display of regulatory actin proteins by cadmium was also reported by Xiao et al. [65]. The downregulated DAAM1 gene asserts the extent to which accumulated testicular cadmium during development can impair gene expression. Waisberg et al. reported that Cd can modulate gene expression, and influence transcription factors and transduction signals [66]. Testicular loss of actin is manifested in germ cell desquamation, enhanced apoptosis, loss of cell polarity, and increased percentage of abnormal sperm [65]. The downregulated pubertal (PND 90) DAAM1 gene in our sturdy further affirms the earlier suggestion, that testicular actin is the target of cadmium toxicity [25], which is in line with the new insight on the mechanism of testicular-induced toxicity by cadmium as documented [65, 67]. The mechanism that alters sperm quality due to cadmium intoxication is still being investigated, but altering proper actin function provided by the inhibited DAAM1 proteins results to testicular damage and premature depletion of germ cells of the seminiferous epithelium, whereby reducing the sperm count [25]. However, the downregulated DAAM1 gene in our study which correlated positively to a reduction in sperm count and enhanced morphological abnormalities in pubertal males at (PND90) was mitigated significantly by co-treatment of the dam with PREOG during gestation and lactation.

In recent times, studies have shown that 50% of infertile males have asthenospermia [68], and efficient motility of spermatozoa is inevitable for it to pass through the female reproductive tract to achieve fertilization [69, 70]. Radial spoke head 6 homolog (RSPH6A) is a testes-specific protein that abounds in the tail of mature sperm cells; thus, it is associated with flagella motility [71]. RSPH6A gene is exclusively expressed in germ cells of the testes during meiosis, and the proteins are part of the cytoskeleton of mature sperm cell flagella in mouse and human [72]. In this study, RSPH6A gene expression in adult male rats (PND90) exposed to cadmium during gestation and lactation was significantly downregulated in the cadmium-treated group, which correlated positively to the decreased sperm motility we reported earlier. This finding is in consonance with what was documented by Romano et al. who reported a significant reduction in RSPH6A in the testes and spermatozoa after 40 days of cadmium treatment [73]. This result further asserts the lethal effects of cadmium, that it can impair testicular functions at different levels, such as the cytoskeleton of cells by comprising the seminiferous epithelium [17, 29, 74], steroidogenesis [20, 75], blood testes barrier [19, 76] and sperm parameters especially motility [76, 77]. However, the co-treatment of the dam with PREOG abrogated the cadmium effect and significantly upregulated the RSPH6A gene in pubertal males (PND90), there was also a corresponding increase in sperm motility.

PTMA is a polypeptide of mammals that is acidic and is associated with the post-meiotic progression of germ cells. It was hypothesized, that PTMA is involved in ensuring a successful acrosome reaction as a result of the role it plays in the interaction between oocyte/spermatozoa and the decondensation of male pronuclear chromatin [78], IAM38 however, is a receptor of inner acrosome membrane that mediate secondary binding of spermatozoa to oocyte and zona pellucida penetration [79]. It was documented by [77] that in vitro exposure to cadmium decreases the protein level of PTMA and IAM38 in the head of spermatozoa, thus impairing its ability to perform acrosome reaction. In this study, we assay for the gene that encodes PTMA protein in the testes of pubertal male rats (PND90) of dam exposed to cadmium during gestation and lactation, and we observed a significant downregulation of the PTMA gene in the cadmium group compared to the control. One can therefore hypothesize, that the downregulated PTMA gene in our study due to maternal exposure to cadmium could also impact IAM38 gene expression as reported in an In vitro study by [77]. consequently, impairing the ability of spermatozoa produced in this testis to perform acrosome reaction, thus reducing male fertility rates. Nevertheless, the co-treatment of the dam with PREOG during gestation and lactation abrogated the cadmium effects and significantly upregulated the PTMA gene in the testes of adult males at (PND90).

Several scientific reports have shown that exposure to environmental toxins in men and animals is life lifelong process, even the embryonic and fetal period is not spared, consequently the manifestation of subfertility/infertility is inevitable in their reproductive age [80]. Cadmium which is one of these chemicals, has been shown to impair testicular functions by mimicking certain endogenous hormones thus disrupting normal endocrine processes (endocrine-disrupting compound (EDC [81] In our study, testosterone value in male offspring (PND90) was significantly decreased compared to the control. We also recorded a significant decrease in GnRH value in the male offspring (PDN90). Furthermore, the LH value was also decreased in this study, but the FSH value significantly increased in the cadmium-treated group compared to the control. Cadmium possesses the ability to alter pituitary hormone secretion by impairing the neurotransmitters that regulate the mechanism [82]. The decrease in GnRH, and LH values in this study and the corresponding increase in FSH value, is an indication that cadmium altered the circadian rhythm in male offspring (PND90) similar to what was documented by [82–84], the decreased in serum testosterone and GnRH values in our study is similar to what was reported by [26]. We also recorded a decrease in the serum progesterone level in the male offspring (PND90) of the dam exposed to cadmium during gestation and lactation. This aligns with what was documented [26]. Nonetheless, the cadmium effects on the testosterone (T), gonadotropin-releasing hormone (GnRH), and progesterone (P_4_) values in the male offspring (PND90) were significantly averted by co-treating of the dam with PREOG during gestation and lactation. Furthermore, the testicular StAR, 3β-HSD and 17β-HSD steroidogenic genes in the male offspring (PND90) investigated in this study, were significantly downregulated by the cadmium treatment and this corresponded to a significant decrease in serum testosterone value. Similar findings of downregulated steroidogenic genes by cadmium were documented [26, 27, 57]. Surprisingly, the cadmium effect on the steroidogenic genes was significantly abrogated in male offspring (PND90) of dam co-treated with PREOG, and the StAR, 3β-HSD and 17β-HSD steroidogenic genes were equally upregulated.

The prepubescent period is very vital in the reproductive health of animals at puberty. This period depends extremely on androgen; thus, any impediment posed by a substance with endocrine-disrupting capability could be detrimental to animal fertility [85]. Early exposure to endocrine-disrupting chemicals during the pertinent developmental period elicits prolonged phenotypic and physiological changes that can be seen only at puberty [86]. In this study, we reported impaired reproductive function in male offspring (PND90) of dam exposed to cadmium during gestation and lactation; such as downregulation of steroidogenic genes which correspond to decreased testosterone level, downregulation of genes associated with the cytoskeleton, motility, and maintenance of normal acrosome membrane integrity which correspond to the decrease sperm motility, viability and enhanced semen morphological abnormality in the cadmium treated group. Also, there was upregulation of oxidative stress markers and corresponding downregulation of testicular antioxidant value in this study. All these findings culminate in a decrease in the reproductive potential of the male offspring (PND90). To ascertain if the decreased reproductive potential translates to decreased reproductive output, a fertility study was done as the final reproductive endpoint to test the ability of males (PND90) exposed to cadmium during gestation and lactation to sire offspring. The male offspring (PND90) that cohabited with the untreated females, had a significant decrease in fertility index, parturition index, and gestation index compared to the control. There was also a significant decrease in the number of pups and the pup birthweight by cadmium treatment compared to control. However, the survival index was insignificant in this study. From our fertility result, we can hypothesize that the decrease testosterone value reported earlier could decrease the libido of the male (PND90) consequently impairing the male fertility index (Table 6). A similar finding of decreased libido was reported [54] because testosterone is indispensable for copulatory feedback to female rodents by its male counterpart [87]. Additionally, the decreased fertility, gestation, and parturition index, number of pups, and pup birthweight could be a result of deteriorating spermatozoa quality/quantity of the male offspring (PND90) due to early cadmium exposure (Table 4) and this could result in pre/post-implantation losses. Which aligns with what was documented by [54]. Khouri and El-Akawi. also reported that decreased reproductive potential from poor sperm quality/quantity can result in pre-implantation [88]. Pre/post-implantation losses have been associated with exposure to toxic substances as these substances can elicit mutagenic or non-mutagenic changes, thus impairing the fertilization process. Non-mutagenic response that can result in pre-implantation loss includes; insufficient mobile/normal sperm, and impaired sperm transport/capacitation reaction [89]. The impaired reproductive potential in the male offspring (PND90) of dam exposed to cadmium during gestation and lactation contributed to the disrupted fertility outcome of male offspring (PND90) observed in this study. However, the impediment posed by cadmium was abrogated significantly by the co-treatment of the dam with PREOG during gestation and lactation, and the reproductive outcome was enhanced (Table 6).

In conclusion, PREOG enhanced the reproductive capacity in male rats (PND90) exposed to cadmium during gestation and lactation by increasing GSI, and EPI of the testes, by enhancing expression of StAR, 3β-HSD and 17β-HSD steroidogenic genes which correspond with the increase in testosterone value in the male offspring (PND90). PREOG also enhances spermatogenesis which corresponds with the upregulated testicular PTMA, RSPH6A, and DAAM1 gene expression in the male (PND90) offspring. PREOG also impaired testicular oxidative stress by downregulating GSH, H_2_O_2_, and MDA in the testes at the same time upregulated testicular antioxidant capacity (SOD & GST) and GPx values in male (PND90) offspring. PREOG impaired the infertility of male offspring (PND90) by cadmium treatment and enhanced the fertility outcome. Further study should be done with extended semen to ascertain the protective role of PREOG and any of the assisted reproductive techniques (ART) can be employed for fertility tests.

## Supplementary Information

Below is the link to the electronic supplementary material.

Supplementary file1 (PPTX 492 KB)

## Data Availability

Primary data is available from the authors upon reasonable request.

## Abbreviations

PREOG: Polyphenol rich extract of Ocimum gratissimum
DAAM1: Disheveled-associated activator of morphogenesis 1
RSPH6A: Radial spoke head 6 homolog A
PTMA: Prothymosin α
3β-HSD1: 3β-Hydroxysteroid dehydrogenase type 1
17β-HSD: 17β-Hydroxysteroid dehydrogenase type 3
StAR: Steroidogenic acute regulatory protein
PND: Postnatal day
CdCL: Cadmium chloride
GSI: Gonadosomatic index
EPI: Epididymal index
SOD: Superoxide dismutase
GST: Glutathione S-transferase
GPx: Glutathione peroxides
MDA: Malondialdehyde
H2O2: Hydrogen peroxide
GSH: Glutathione
q-RT-PCR: Quantitative real time polymerase chain reaction
ROS: Reacting oxygen specie
DNA: Deoxyribonucleic acid
AR: Acrosome reaction
OS: Oxidative stress
FSH: Follicle stimulating hormone
LH: Luteinizing hormone
T: Testosterone
GnRH: Gonadotropin realizing hormone
