# Supplementary material for: The Complicity of DAAM1, PTMA, RSPH6A, and Steroidogenic Genes in the Fertility of Male Rats Exposed to Cadmium During Gestation and Lactation: Attenuation by PREOG
Source: Reprod Sci. 2025 Jul 8;32(8):2742–62. doi: 10.1007/s43032-025-01902-x (PMC12361343; doi:10.1007/s43032-025-01902-x)
Supplement: Supplementary file 1 — Supplementary file1 (PPTX 492 KB) [file 43032_2025_1902_MOESM1_ESM.pptx]

## Slide 1
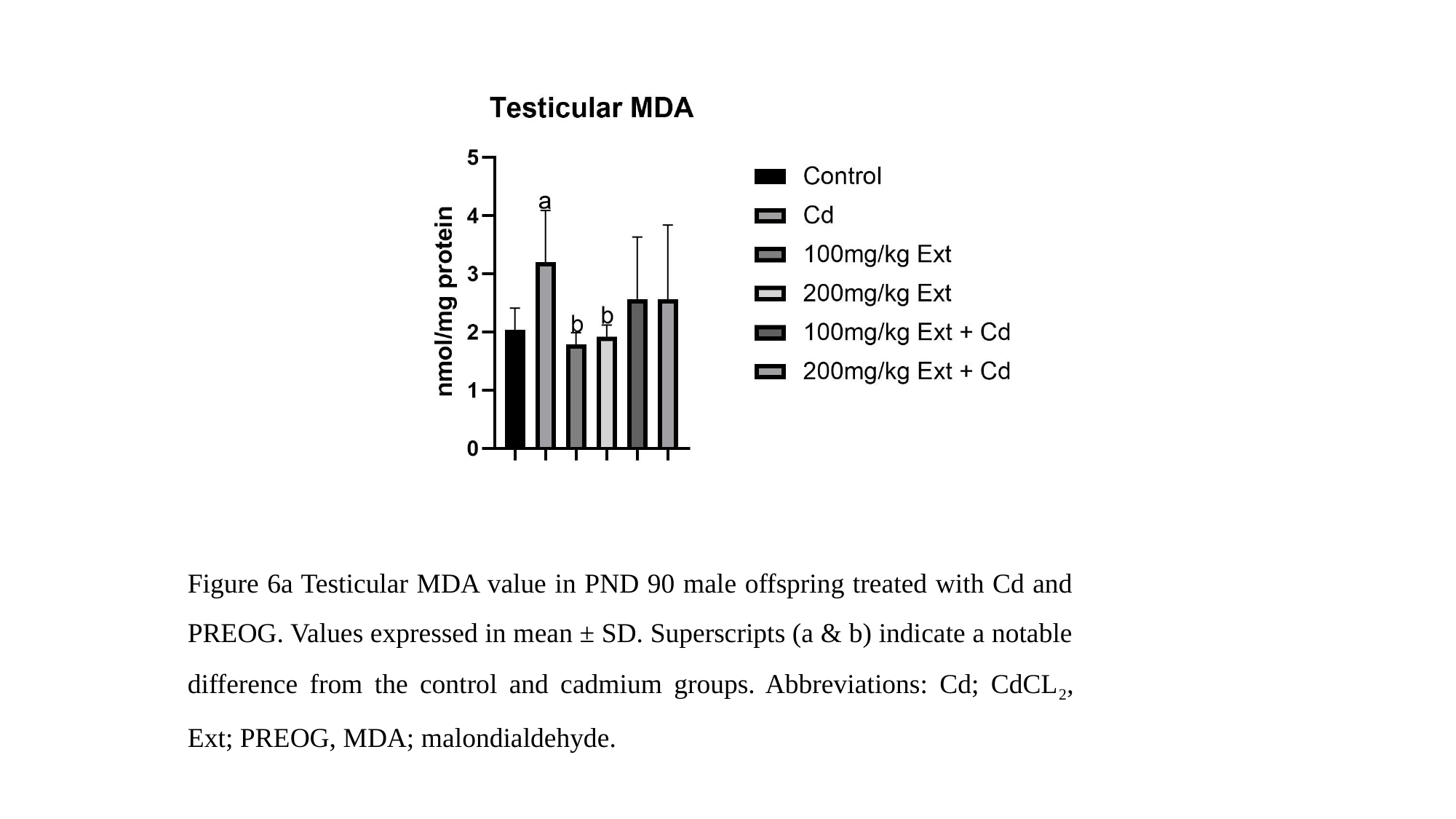

Figure 6a Testicular MDA value in PND 90 male offspring treated with Cd and PREOG. Values expressed in mean ± SD. Superscripts (a & b) indicate a notable difference from the control and cadmium groups. Abbreviations: Cd; CdCL2, Ext; PREOG, MDA; malondialdehyde.

## Slide 2
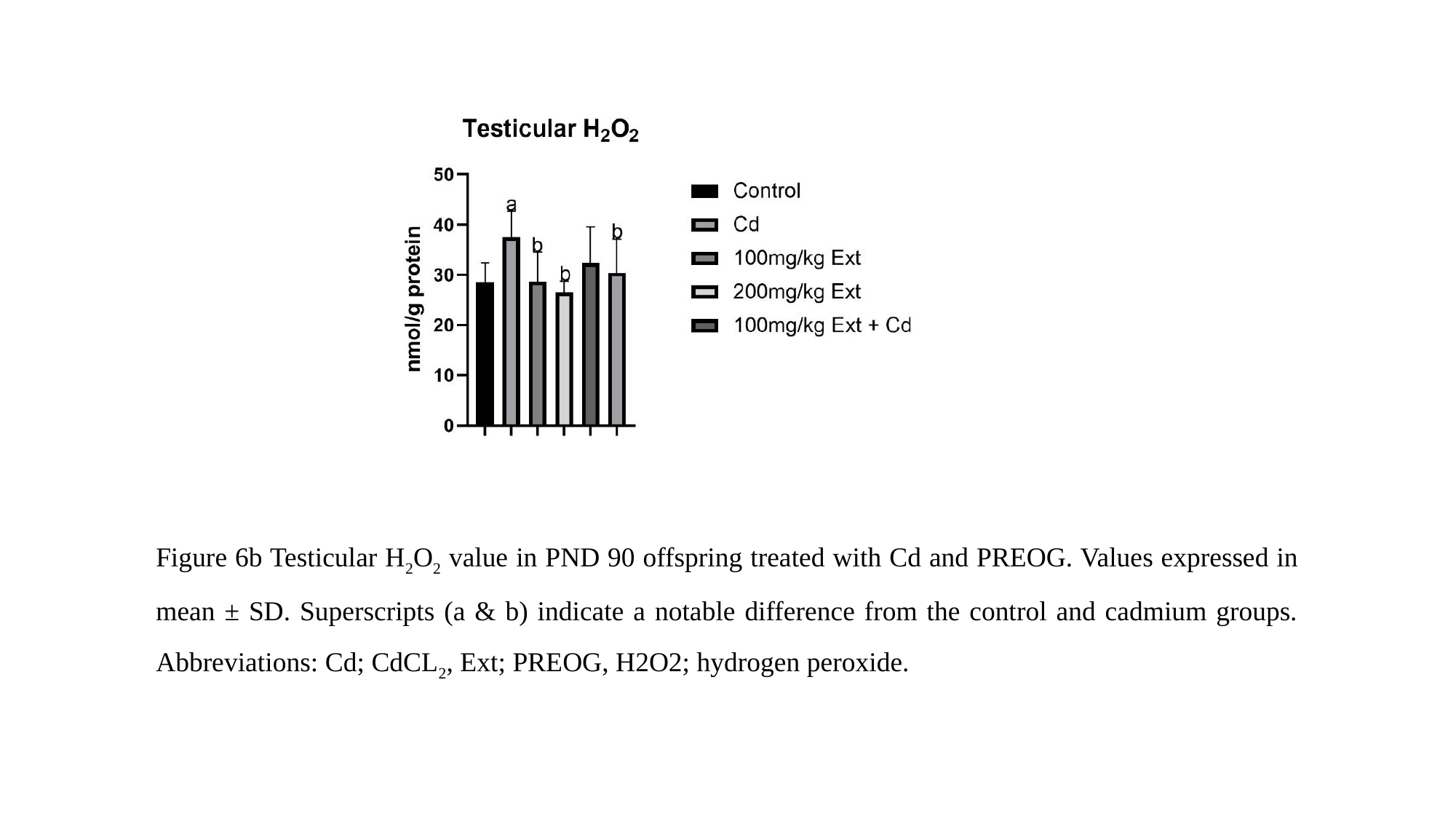

Figure 6b Testicular H2O2 value in PND 90 offspring treated with Cd and PREOG. Values expressed in mean ± SD. Superscripts (a & b) indicate a notable difference from the control and cadmium groups. Abbreviations: Cd; CdCL2, Ext; PREOG, H2O2; hydrogen peroxide.

## Slide 3
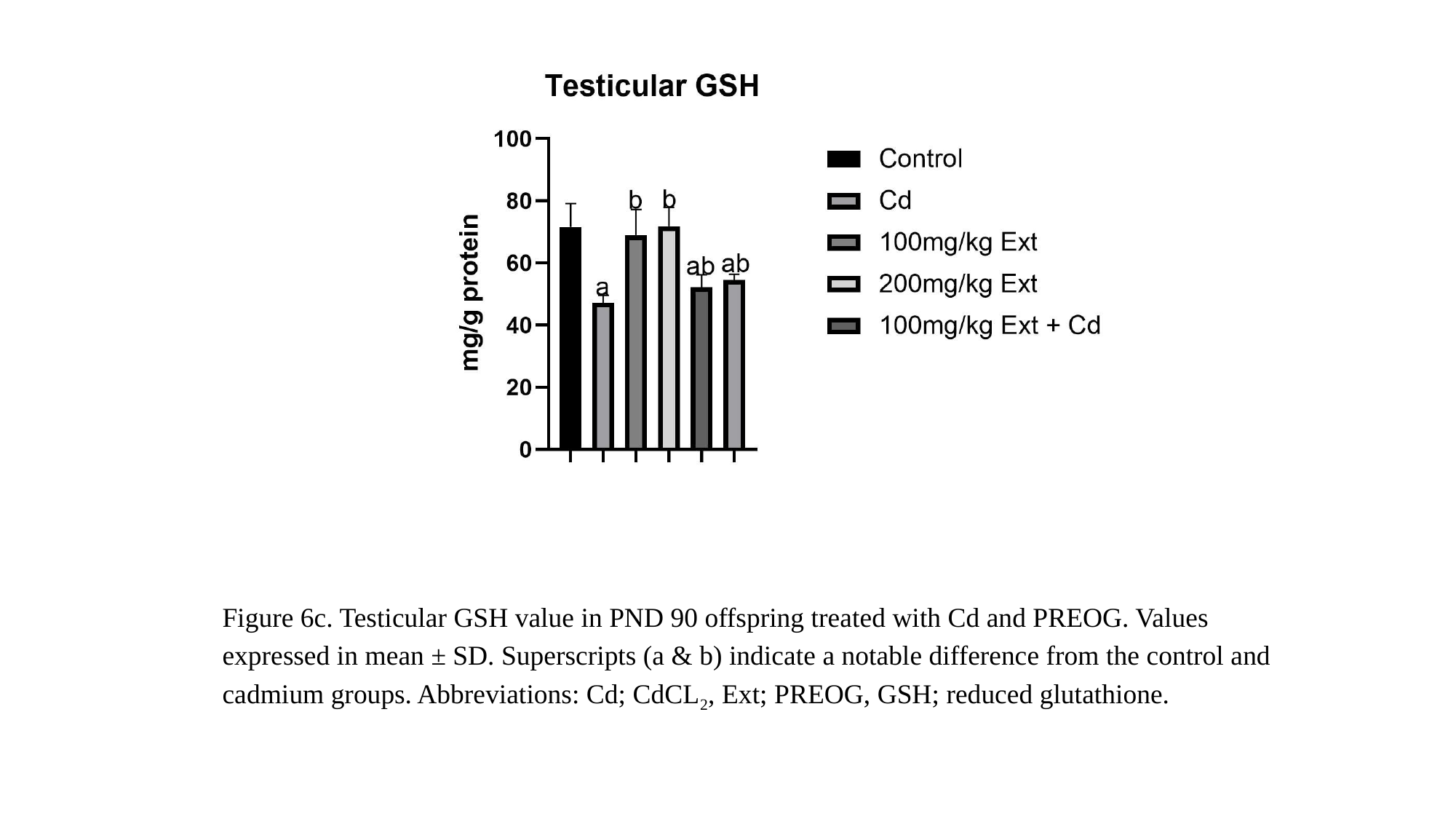

Figure 6c. Testicular GSH value in PND 90 offspring treated with Cd and PREOG. Values expressed in mean ± SD. Superscripts (a & b) indicate a notable difference from the control and cadmium groups. Abbreviations: Cd; CdCL2, Ext; PREOG, GSH; reduced glutathione.

## Slide 4
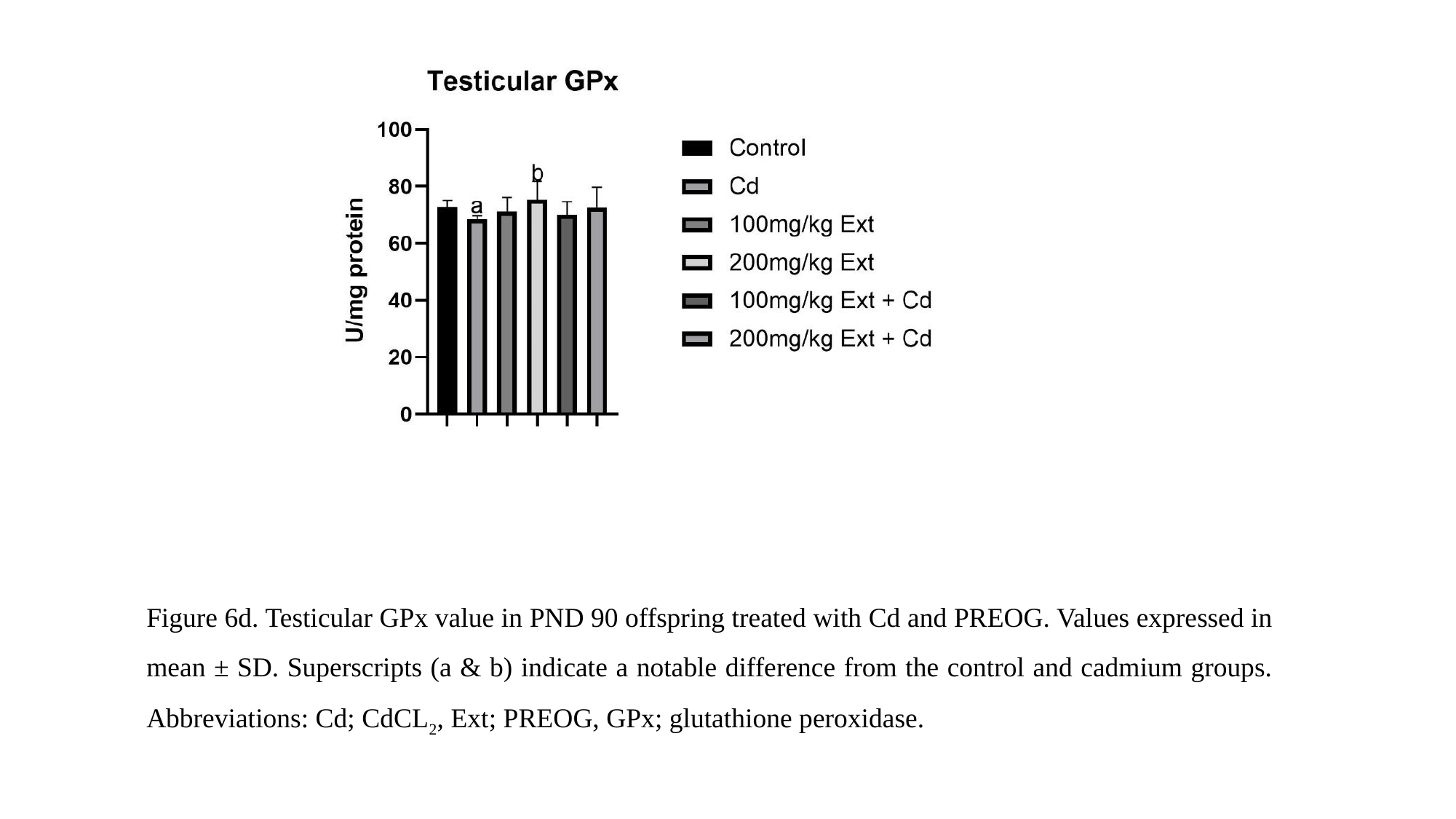

Figure 6d. Testicular GPx value in PND 90 offspring treated with Cd and PREOG. Values expressed in mean ± SD. Superscripts (a & b) indicate a notable difference from the control and cadmium groups. Abbreviations: Cd; CdCL2, Ext; PREOG, GPx; glutathione peroxidase.

## Slide 5
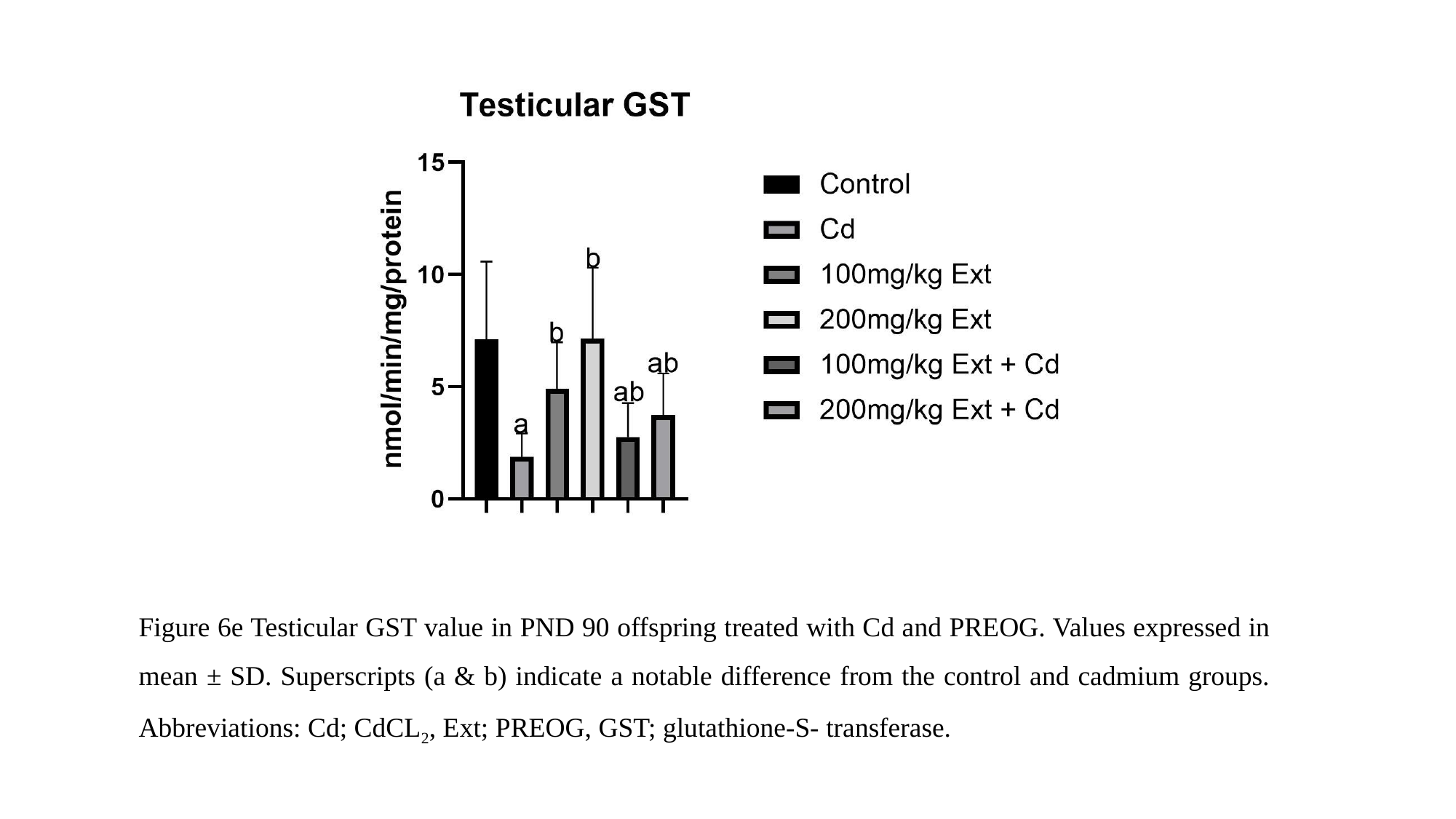

Figure 6e Testicular GST value in PND 90 offspring treated with Cd and PREOG. Values expressed in mean ± SD. Superscripts (a & b) indicate a notable difference from the control and cadmium groups. Abbreviations: Cd; CdCL2, Ext; PREOG, GST; glutathione-S- transferase.

## Slide 6
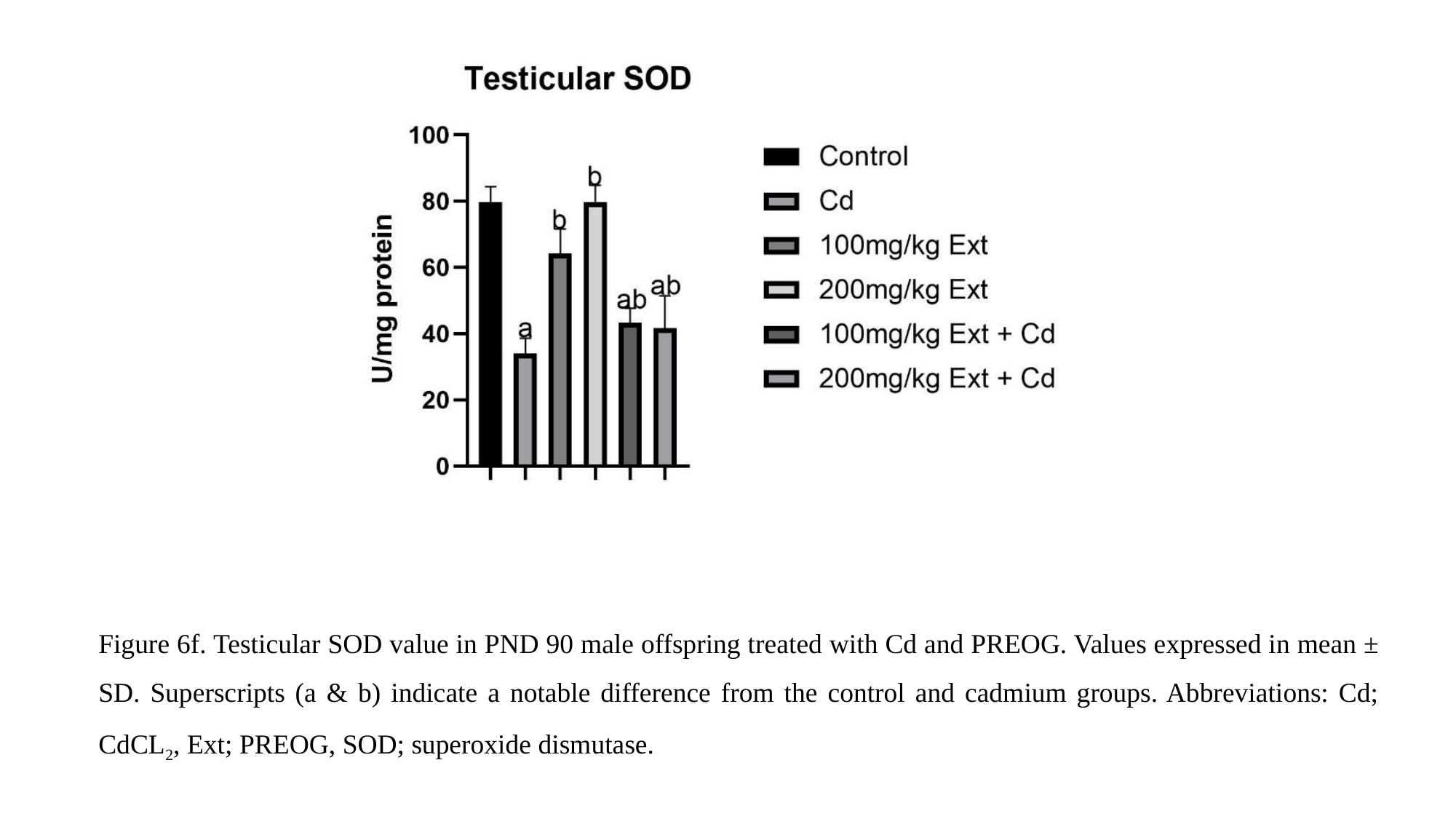

Figure 6f. Testicular SOD value in PND 90 male offspring treated with Cd and PREOG. Values expressed in mean ± SD. Superscripts (a & b) indicate a notable difference from the control and cadmium groups. Abbreviations: Cd; CdCL2, Ext; PREOG, SOD; superoxide dismutase.
